# Targeting *TROY*-mediated P85a/AKT/TBX3 signaling attenuates tumor stemness and elevates treatment response in hepatocellular carcinoma

**DOI:** 10.1186/s13046-022-02401-6

**Published:** 2022-05-25

**Authors:** Beilei Liu, Xiaona Fang, Dora Lai-Wan Kwong, Yu Zhang, Krista Verhoeft, Lanqi Gong, Baifeng Zhang, Jie Chen, Qianqian Yu, Jie Luo, Ying Tang, Tuxiong Huang, Fei Ling, Li Fu, Qian Yan, Xin-Yuan Guan

**Affiliations:** 1 Department of Clinical Oncology, The University of Hong Kong-Shenzhen Hospital, Shenzhen, China; 2 Department of Clinical Oncology, The University of Hong Kong, Hong Kong, China; 3 State Key Laboratory for Liver Research, The University of Hong Kong, Hong Kong, China; 4 State Key Laboratory of Oncology in Southern China, Sun Yat-Sen University Cancer Center, Guangzhou, China; 5 School of Chinese Medicine, The University of Hong Kong, Hong Kong, China; 6 Ruijin Hospital, Shanghai Jiaotong University School of Medicine, Shanghai, China; 7 Department of Pharmacology and International Cancer Center, Department of Orthopedics, Shenzhen University Health Science Center, Shenzhen, China; 8 School of Bioscience and Bioengineering, South China University of Technology, Guangzhou, China; 9 Guangdong Provincial Key Laboratory of Colorectal and Pelvic Floor Diseases, Guangdong Institute of Gastroenterology, The Sixth Affiliated Hospital, Sun Yat-Sen University, Guangzhou, China; 10 Advanced Energy Science and Technology Guangdong Laboratory, Huizhou, China; 11 MOE Key Laboratory of Tumor Molecular Biology, Jinan University, Guangzhou, China

**Keywords:** Oncofetal properties, Pluripotency signaling pathway, K63 polyubiquitin modification, Cancer-associated fibroblast

## Abstract

**Background:**

Previous in vitro hepatocyte differentiation model showed that TROY was specifically expressed in liver progenitor cells and a small proportion of hepatocellular carcinoma cells, suggesting that TROY may participate in hepatocellular carcinoma (HCC) stemness regulation. Here, we aim to investigate the role and mechanism of TROY in HCC pathogenesis.

**Method:**

Bioinformatics analysis of the TCGA dataset has been used to identify the function and mechanism of TROY. Spheroid, apoptosis, and ALDH assay were performed to evaluate the stemness functions. Validation of the downstream pathway was based on Western blot, co-immunoprecipitation, and double immunofluorescence.

**Results:**

HCC tissue microarray study found that a high frequency of TROY-positive cells was detected in 53/130 (40.8%) of HCC cases, which was significantly associated with poor prognosis and tumor metastasis. Functional studies revealed that TROY could promote self-renewal, drug resistance, tumorigenicity, and metastasis of HCC cells. Mechanism study found that TROY could interact with PI3K subunit p85α, inducing its polyubiquitylation and degradation. The degradation of p85α subsequently activate PI3K/AKT/TBX3 signaling and upregulated pluripotent genes expression including SOX2, NANOG, and OCT4, and promoted EMT in HCC cells. Interestingly, immune cell infiltration analysis found that upregulation of TROY in HCC tissues was induced by TGF-β1 secreted from CAFs. PI3K inhibitor wortmannin could effectively impair tumor stemness to sorafenib.

**Conclusion:**

We demonstrated that TROY is an HCC CSC marker and plays an important role in HCC stemness regulation. Targeting TROY-positive CSCs with PI3K inhibitor wortmannin combined with chemo- or targeted drugs might be a novel therapeutic strategy for HCC patients.

**Graphical abstract:**

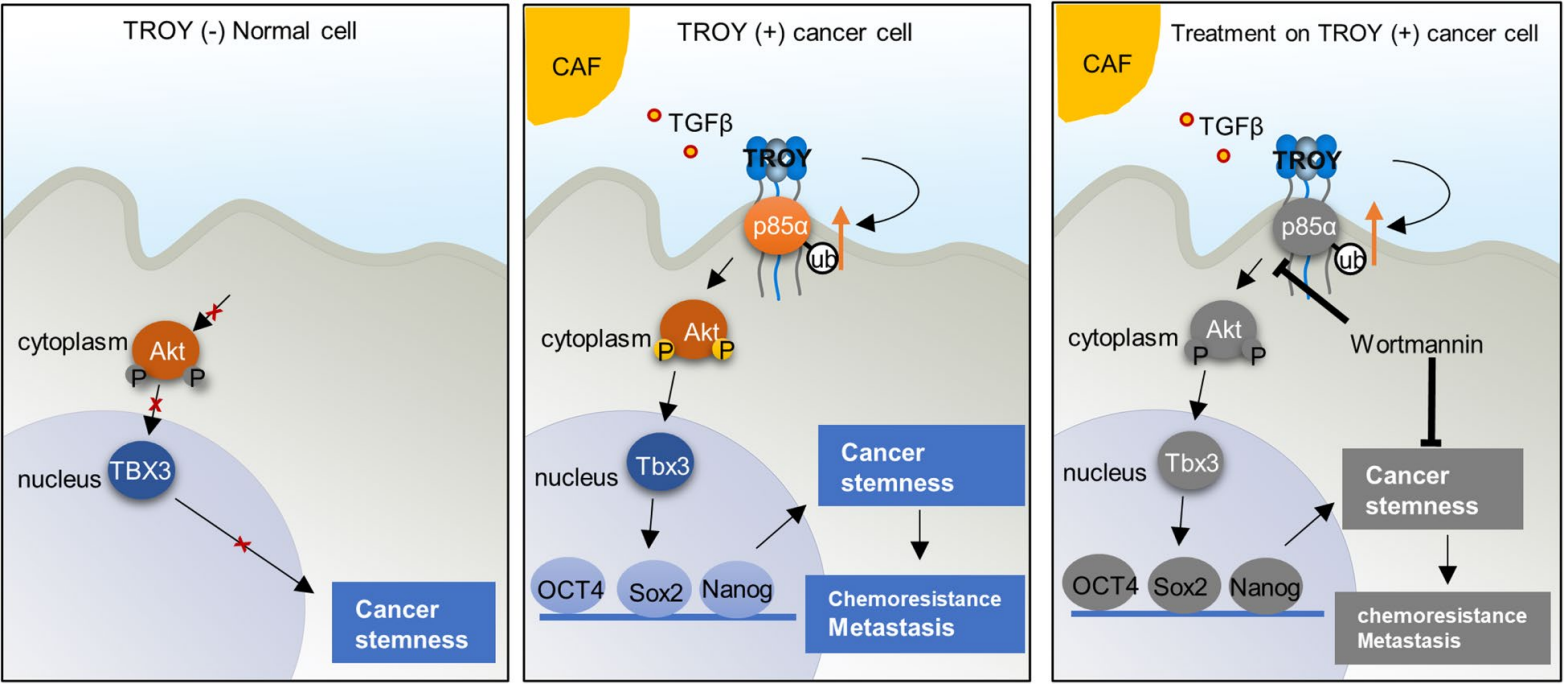

**Supplementary Information:**

The online version contains supplementary material available at 10.1186/s13046-022-02401-6.

## Background

Hepatocellular carcinoma (HCC) is the sixth malignant cancer and the fourth cause of cancer death worldwide in 2018 [1]. Lack of suitable biomarkers for early diagnosis and limited treatment strategies for cancer recurrence, metastasis, and chemoresistance are the main reasons for high mortality in liver cancer. Cancer stem cells (CSCs) are a minor subpopulation of cancer cells sharing similar characteristics with tissue progenitor cells such as capabilities of self-renewal and multi-lineage differentiation, which play important roles in cancer recurrence and metastasis [2–4]. Signaling pathways responsible for normal stem cells development were reported to be hijacked by tumor cells, such as c-MYC, TGF-β, Notch, Hippo, Wnt, and Hedgehog signaling [5]. Clinically, poor-differentiated HCCs with high recurrence rates always expressed markers of liver progenitors including alpha-fetoprotein (AFP), cytokeratin 19 (CK19), cytokeratin 7 (CK7), and SRY-Box Transcription Factor 9 (SOX9) [6–9]. All these findings suggest that understanding the regulatory network of liver progenitor cells is important to identify CSCs targets in HCC.

We previously established an in vitro hepatocyte differentiation model, of which human embryonic stem cells (ES) were induced to be differentiated into definitive endoderm (DE), liver progenitor (LP) cells, and premature hepatocytes (PH) [10]. By deep RNA-sequencing, we identified a previously undescribed gene expression pattern of liver progenitor cells, which might be important to HCC progression. Bioinformatic-aided network analysis has identified several novels oncofetal drivers of HCC, of which, a liver progenitor cell-specific gene, TNF Receptor Superfamily Member 19 (*TNFRSF19*), also known as *TROY*, was investigated in this study. *TROY* belongs to the TNF receptor superfamily, which normally transduces signals upon ligand interaction via specific adaptor proteins bound to their intracellular domain [11]. However, TROY is different because no such ligands have been identified to interact with TROY, and its intracellular adaptor proteins remain to be elusive [12, 13]. Functionally, TROY was reported to be a stem cell marker in kidney [14], brain [11], and gastric units [15]. High expression of *TROY* is associated with poor prognosis in colorectal cancer [16], glioblastoma [17], and nasopharyngeal carcinomas [18]. However, the precise role and underlying mechanism of TROY in liver CSCs have not been explored.

In this study, we characterized TROY as a novel CSC marker of HCC. Overexpression of *TROY* could increase HCC stemness both in vitro and in vivo. Mechanically, *TROY* was found to activate PI3K/AKT/TBX3 pathway via direct protein–protein interaction with PI3K subunit p85α. PI3K/AKT/TBX3 axis plays an important role in maintaining pluripotency of mouse ES cells [19] and it has been reported as a treatment target in multi type of embryonal cancers [20]. As an essential protein for pluripotency maintenance, TBX3 transcriptionally upregulates expressions of core pluripotent genes SOX2, NANOG, OCT4 and it can promote cell metastasis via activating the EMT pathway in HCC cells. The roles of TROY in liver CSCs might provide potential therapeutic targets for HCC treatment.

## Methods

### HCC samples and cell lines

HCC tissue microarray (TMA) containing 148 pairs of primary HCC specimens and their corresponding non-tumor tissues were constructed as described previously [21]. All samples were collected from Sun Yat-sen University Cancer Center (Guangzhou, China) between 2003 and 2010. Human HCC cell lines Huh7, HepG2, SNU398, SNU182, SNU878, and human embryonic kidney cell line 293FT were purchased from American Type Culture Collection (ATCC, Manassas, Virginia, USA). Immortalized human hepatocyte cell line LO2 was obtained from the Institute of Virology. Cell lines Huh7, HepG2, LO2, and 293FT were cultured in DMEM (high glucose) supplemented with 10% fetal bovine serum (FBS). SNU398, SNU182, SNU878 were maintained in RPMI-1640 medium with 10% FBS. HepG2 was cultured in MEM containing 10% FBS. All cell lines were incubated at 37 °C and 5% CO_2_.

### Construction of TROY Overexpression and knockdown cells

Briefly, the full-length *TROY* isoform1 (NM_018647.4) and isoform2 (NM_148957.3) were cloned into the pLenti6 expression vector (Invitrogen) separately. Lentiviral shRNA clones for *TROY* were obtained from GeneCopoeia (HSH106281-LVRU6GP for *TROY* and CSHCTR001-LVRU6GP for a scramble control) targeted both isoform 1 and 2. Stable knockout *p85α* HCC cell line by lentiviral transduction of *p85α* single guide RNA (Supplementary Table 4) lentiCRISPR v2. For lentivirus production, HEK293T packaging cells were transfected with lentiviral vectors using Lipofectamine 2000 Reagent (Invitrogen). After 72 h of incubation, the viral supernatant was collected and filtered. HCC cells were incubated overnight with the viral supernatant and supplemented with 10 μg/ml polybrene. Stable clones were selected using puromycin.

### QPCR and western blotting

Total RNA was extracted via TRIZOL Reagent (Invitrogen), and cDNA was synthesized by a reverse transcription PCR kit (TAKARA). Quantitative real-time PCR (qPCR) was performed using the TB Green Premix Ex Taq II (TAKARA) and a LightCycler® 480 System (ROCHE). The mRNA expression of *TROY, NANOG, SOX2, OCT4, TBX3,* and *β-Actin* was assessed by qPCR with the primers (Supplementary Table 5). Total RNA was extracted using TRIZOL. The cDNA was synthesized from 0.5 μg of total RNA by PrimeScript RT Master Mix (Takara Bio). The protein expression of genes in this research was assessed by western blotting (Supplementary Table 5).

### In vitro cell proliferation, drug resistance and apoptosis assays

In vitro cell proliferation rate was assessed by monitoring the cell number for 5–7 days in cell culture. The cells in each condition were seeded at 1,000 cells/well for proliferation assay or 8,000 cells/well for drug resistance assay in 96-well in triplicate and incubated in a 37 °C humidified CO_2_ incubator with the drug or vehicle containing medium refreshed every other day. The number of cells was determined by XTT kit (Roche Diagnostics, Indianapolis, IN). For the apoptosis assay, cells were seeded at 5 × 10^5^ cells/well into a six-well plate in triplicate. After 24 h, cells were subjected to the drug or vehicle containing medium for 24–48 h and then followed by flow cytometry analysis. The cell apoptosis assay was determined according to the manual of PE Annexin V Apoptosis Detection Kit I (BD Biosciences). Data were performed by FlowJo software.

### ALDH measurement

To determine the intracellular aldehyde dehydrogenase (ALDH) concentration, an equal number of HCC cells were seeded onto six-well dishes. The attached cells were trypsinized and washed with PBS. Cells were stained with AldeRed™ ALDH Detection Assay (Sigma-Aldrich SCR150) for analyzing the ALDH level. Stained cells were analyzed using the FACSCanto II Analyzer (BD Biosciences). The results were performed with FlowJo software.

### In vitro spheroid formation, migration, and invasion assay

Sphere-formation assay is an in vitro method commonly used to identify CSCs and study their self-renewal properties. For spheroid formation, a total number of 3,000 cells were seeded in Corning® Costar® Ultra-Low Attachment Multiple 6-Well Plate (Merck, CLS3471) with DMEM/F12 medium (Life Technologies), supplemented with 20 ng/mL human recombinant EGF (Life Technologies), 10 ng/mL human recombinant FGF (Life Technologies), B27 (1: 50, Invitrogen), and 4ug/mL insulin (BIOIND, Kibbutz Beit Haemek, Israel) in triplicate and incubated in a 37 °C humidified CO2 incubator with 30 uL medium refreshed every second day. For serial passage of spheroids, the primary spheroids were centrifuged and dissociated to single cells using TrypLE Expression (Invitrogen) and neutralized the reaction using trypsin inhibitor (Invitrogen).

Migration and invasion assays were determined to evaluate cell motility (BD Biosciences, San Jose, CA). Approximately 8 × 10^4^cells were seeded in the medium without FBS on transwell upper chambers, and the lower chamber was supplied with a medium of complete 10% FBS. After 24-48 h, the lower membrane surface was stained using crystal violet to count the cell number under a microscope.

### In vivo* functional assay*

For the subcutaneous injection model, 5 × 10^6^ *TROY*-transfected cells, sh*TROY*-transfected cells, and their corresponding control cells were injected subcutaneously into different sides of 4-week-old BABL/c nude mice or NOD/SCID mice. The tumor size was measured every 5–7 days with a caliper and calculated with the formula 0.5 × length × width × width. Mice were sacrificed 4–6 weeks. For the metastasis mice model, 1 × 10^6^ *TROY*-transfected cells, sh*TROY*-transfected cells, and their corresponding control cells were injected through the tail vein of BABL/c nude mice 6-week-old. Mice were sacrificed around 70 days. For drug treatment, 2 weeks after tumor implantation, mice were administered with (1) vehicle with DMSO, (2) 20 mg/kg sorafenib every day via oral gavage, (3) 1 mg/kg wortmannin every day via oral gavage, (4) combine treatment. After 5 weeks, mice were sacrificed.

### Limiting dilution assay

HCC transfected TROY and vector cells or shTROY and shNTC cells were subcutaneously implanted 5 × 10^4^, 10 × 10^4^, 10 × 10^5^ and 5 × 10^6^ cells into NOD/SCID mice, respectively. The mice were sacrificed 7 weeks postinoculation, and the CSC proportions were analyzed as described.

### Isolation of cancer-associated fibroblasts

Briefly, freshly collected HCC tumour tissue were cut into as small pieces as possible in sterile phosphate-buffered saline (PBS) solution, followed by collagenase digestion. The suspension was filtered through 20 mm stainless steel wire mesh to collect a single cell suspension. The filtrate was centrifuged and washed before being finally plated on 6 cm tissue culture dishes in 5 ml DMEM medium supplemented with 20% fetal bovine serum (FBS). After culturing for 30 min at 37℃, nonadherent cells (mainly tumor cells) were removed to obtain pure fibroblasts. The adherent fibroblasts were subcultured for further study.

### Conditioned medium

Cancer-associated fibroblasts were cultured in DMEM medium with 10% FBS until 70% confluence. After complete removal of the normal culture medium, CAF cells were continually cultured in DMEM medium with 3% FBS for 24 h before medium collection. The culture medium was then centrifuged at 1000 g for 30 min and the supernatant was collected as the conditioned medium for further study.

### Statistical analysis

Statistical analysis was performed using GraphPad Prism V.8 software. Independent student’s t-test was used to compare parametric continuous variables. Pearson’s χ2 test was performed to compare the correlation between genes and clinicopathological parameters. Kaplan–Meier plots and log-rank tests were used to analyze HCC patient survival. For limiting dilution assay, the tumor-initiating frequency and statistics were calculated with ELDA software. The values represent the mean ± SD of three independent experiments. The P-values were marked as *P < 0.05, **P < 0.001, and ***P < 0.0001 in all figures.

## Results

### TROY is specifically expressed in liver progenitors

**Fig. 1:**
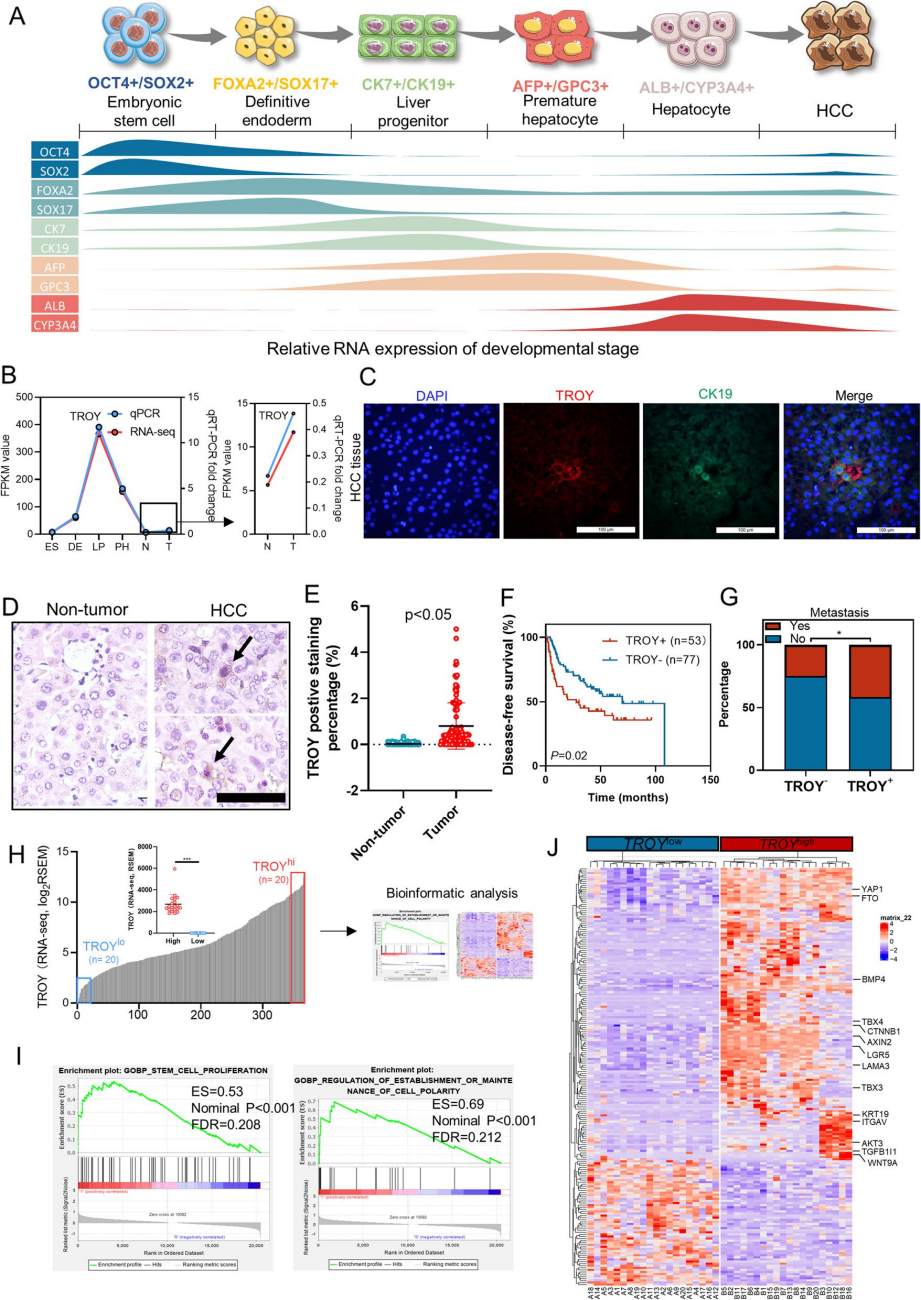
Association of *TROY* expression with liver development and poor HCC survival. (**A**) An in vitro hepatocyte differentiation model. Expression pattern of characteristic markers for ES (OCT4, SOX2), EN (FOXA2, SOX17), LP (CK19, CK7), PH (AFP), and normal liver tissue (ALB, CYP3A4). (**B**) *TROY* expression pattern was verified in the differentiation model and qPCR. (**C**) Representative dual-color immunofluorescence analysis of HCC clinical samples showing the co-localization of CK19(green) and TROY (red), Scale bar = 100 μm. (**D**) Representative IHC staining of *TROY* in non-tumor and HCC tissues. *TROY*^+^ cells were pointed by arrows. (**E**) Comparison of the TROY positive cells between tumor and non-tumor tissues. (**F**) Kaplan–Meier survival curves showed HCC patients with TROY positive staining (*n* = 55) had worse survival outcomes compared with patients without detectable TROY staining (*n* = 77). (**G**) Correlation analyses between *TROY* positive cells and clinical metastasis characteristics in HCCs. (**H**) Comparison of 20 highest *TROY* expression patients and 20 lowest *TROY* expression patients from TCGA database was performed deseq2. (**J**) GSEA analysis of *TROY*^high^ and *TROY*.^low^ groups. (**I**) Heatmap of differentially expressed genes of high and low *TROY* expression groups

Recently, we established an in vitro hepatocyte differentiation model, in which human embryonic stem cells (ES) were induced to differentiate into endoderm (DE), liver progenitor (LP), and premature hepatocytes (PH) [10]. Deep RNA-sequencing was conducted for cells in these four developmental stages, as well as normal liver specimens (N) and HCC clinical samples (T). The genes which mark different hepatocyte developmental stages, including ES markers (*OCT4* and *SOX2*), EN markers (*FOXA2* and *SOX17*), LP markers (*CK19* and *CK7*), PH markers (*AFP* and *GPC3*) and mature hepatocyte markers (*ALB* and *CYP3A4*) showed their unique expression pattern in this model, indicating the validity of this model (Fig. 1A). To identify genes involving stemness regulation, we focused on a cluster of genes with specific expression in liver progenitors (Supplementary Table 1). KEGG pathway and Gene Ontology (GO) enrichment analysis revealed that this cluster of genes was closely associated with the Wnt signaling pathway, the pluripotency of stem cells, and developmental signaling (Supplementary Fig. 1A). Among these genes, *TROY* was selected for further study with its distinct expression pattern. Both RNA-sequencing data and qPCR results revealed that *TROY* reached its peak expression in the liver progenitor stage and dramatically decreased following differentiation but increased in HCC (Fig. 1B). Double staining showed that TROY^+^ cells were co-expressed with hepatic progenitor marker CK19 (Fig. 1C). These findings suggested that TROY may represent and mark specific HCC CSC subsets.

### Overexpression of TROY is correlated with poor outcome

To validate the expression and clinical association of TROY in HCCs, a tissue microarray containing 148 pairs of primary HCCs (tumor *vs* non-tumor tissues) was applied to analyze the association of TROY expression with clinicopathological features. Informative immunohistochemistry (IHC) results were obtained in 130 HCCs. Non-informative samples included lost samples and samples with too few cells. IHC results found that TROY-expressing cells were detected in most HCC tumor tissues with expression percentage around 0–5%, while the expression of TROY is rarely detected in non-tumor tissues (Fig. 1D, 1E). Based on the frequency of TROY positive cells, the HCC patients were divided into high-frequency group (TROY^+^, > 0.5%, *n* = 53) and low-frequency group (TROY^*−*^, ≤ 0.5%, *n* = 77, Supplementary Table 2). Kaplan–Meier survival analysis found that the disease-free survival (DFS) rate was lower in the TROY^+^ group compared with the TROY^−^ group (*P* = 0.02; Fig. 1F). In addition, clinical association study found that TROY expression was significantly correlated with age, tumor size and metastasis (Fig. 1G, Supplementary Table 3).

Since TROY, like other CSCs markers expressed in a minor subpopulation of HCC malignant cells, high mRNA expression of *TROY* represented a high percentage of TROY expression in that patient. Here, bioinformatics-aided analysis based on RNA-sequencing data from the HCC TCGA database to get an in-depth look at whether the expression of TROY was associated with pluripotency signaling in HCC patients. HCC samples were ranked by their expression of *TROY*, and the differentially expressed genes were identified between the top 20 and the last 20 cases with the highest (*TROY*^*hi*^) and lowest (*TROY*^*lo*^) expression (Fig. 1H). Gene set enrichment analysis (GSEA) results showed that high expression of *TROY* is significantly associated with the established gene sets “stem cell proliferation” and “regulation of establishment or maintenance of cell polarity” (Fig. 1I). By using Deseq2 (log_2_ FC ≥ 4, *P* < 0.01) [22], 823 up-regulated genes and 370 down-regulated genes were found. The expression profile of these genes was able to effectively segregate *TROY*^*hi*^ patients and *TROY*^*lo*^ patients in unsupervised clustering analysis. *TROY*^*hi*^ patients exhibit increased expression of various stemness-related genes (Fig. 1J). Collectively, these findings strongly suggest that *TROY* might be involved in regulating stemness-related signaling in HCC.

### TROY enhances stemness propertises of HCC cells

**Fig. 2:**
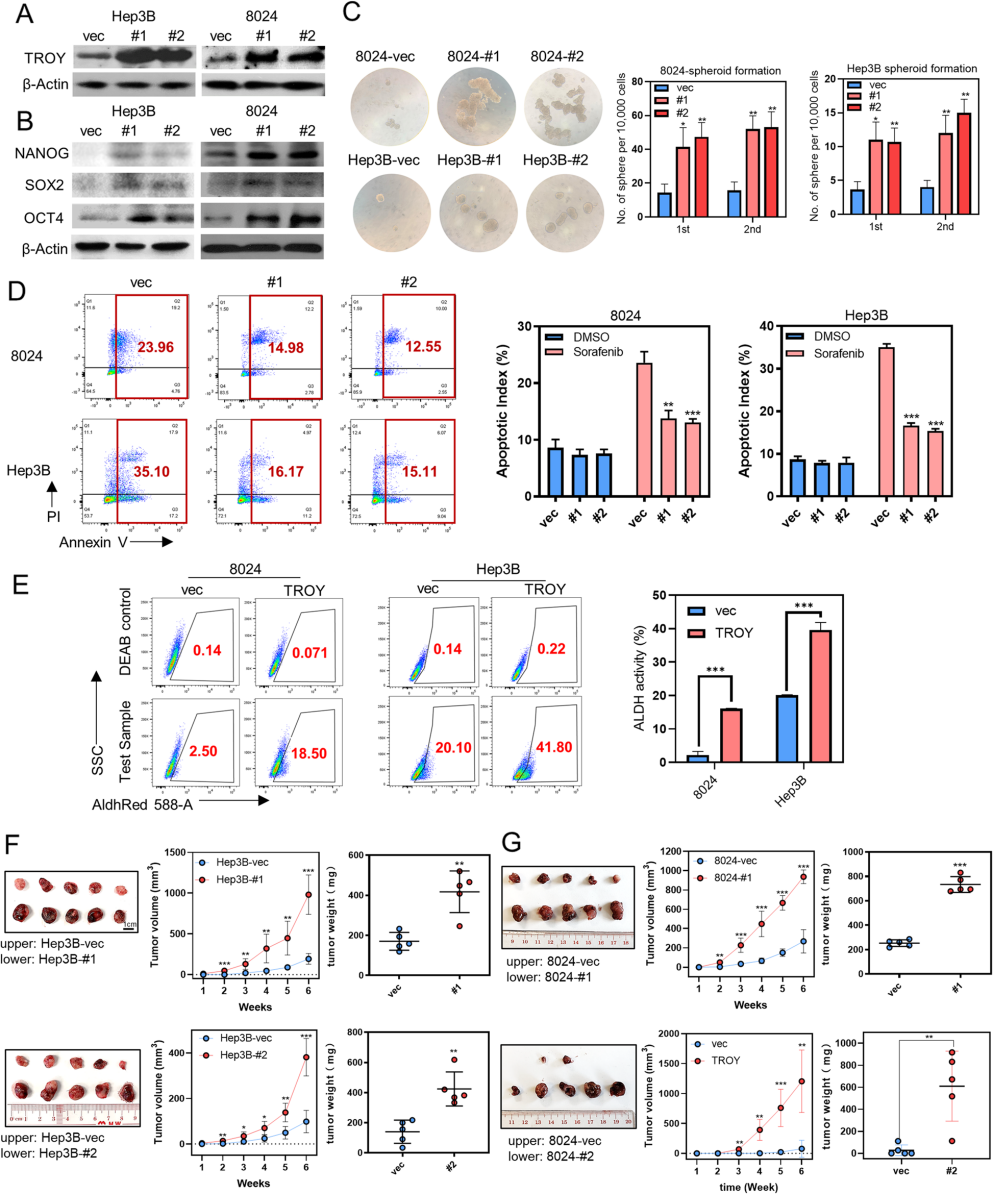
*TROY* enhances the stemness properties of HCC cells. (**A**, **B**) Western blotting of *TROY* (**A**), NANOG, SOX2, and OCT3/4 (**B**) in 8024 and Hep3B HCC cells transfected with lentiviruses expressing empty vector (vec) or *TROY* isoforms (#1 and #2). β-Actin was used as a loading control. (**C**) Spheroid formation assay (left) and summaries (right) of spheroid numbers in 8024 and Hep3B cells with vec or *TROY* isoforms. (**D**) Representative flow cytometry plots (left) and summary (right) of apoptotic cells in 8024 and Hep3B cells transfected with vec or *TROY* isoforms. Cells were treated with Sorafenib (20 μm) for 24 h. (**E**) Representative flow cytometry plots (left) and summary (right) of ALDH activity in 8024 and Hep3B cells transfected with vec or *TROY* isoforms. (**F**) Tumor (left), tumor volume (middle) and tumor weight (right) of in vivo tumor formation assays with subcutaneously injected Hep3B cells transfected with vec or *TROY* isoforms in NOD/SCID mice. (**G**) In vivo subcutaneous implantation nude mice model induced by 8024 cells transfected with vec or *TROY* isoforms. Statistical significances: *, *P* < 0.05; **, *P* < 0.01; ***, *P* < 0.001

*TROY* has 2 major transcripts: *TROY* isoform 1(#1) encodes a 423 amino acid polypeptide and *TROY* isoform 2(#2) encodes a 417 amino acid polypeptide. The amino acid sequence of isoform 2 differs from isoform 1 in position 416–423. To investigate the potential role of *TROY* in cancer stemness maintenance, we firstly checked the expression level of *TROY* in all HCC and immortalized liver cell lines by flow cytometry, qPCR, and western blot analysis. Compared with immortalized liver cell line LO2, all the other HCC cell lines showed upregulated *TROY* expression (Supplementary Fig. 1B, C, D). Then the two major isoforms of *TROY* were separately cloned into a lentiviral vector and stably transfected into Hep3B, PLC8024, and LO2 cells. The ectopic expression of *TROY* was confirmed in both protein and mRNA levels by qPCR (Supplementary Fig. 2A), immunofluorescence (Supplementary Fig. 2B), and western blot (Fig. 2A, Supplementary Fig. 2C). In contrast to control cells, *TROY*-overexpressing cells showed marked upregulation of pluripotency markers such as OCT4, NANOG, and SOX2 expression by western blot (Fig. 2B, Supplementary Fig. 2C), qPCR (Supplementary Fig. 2D), immunofluorescence (Supplementary Fig. 2E). Functionally, the introduction of *TROY* enhanced capabilities of spheroid formation (Fig. 2C, Supplementary Fig. 3A), colony formation (Supplementary Fig. 3B). Also, it accelerated cell cycle progress (Supplementary Fig. 3C, 3D) and cell proliferation (Supplementary Fig. 3E). Since resistance to chemotherapy is an important hallmark of CSCs, we then investigated whether *TROY* confers chemo-resistance features to HCC cells. *TROY-*overexpressing cells demonstrated a lower apoptotic rate (Fig. 2D, Supplementary Fig. 3F) and higher cell viability (Supplementary Fig. 4A-C) in the presence of cisplatin, 5-Fu, or sorafenib treatment. As high ALDH activity leads to several types of malignancies, serves as a cancer stem cell marker, and correlates with poor prognosis [23], we then tested ALDH activity in cells transfected with *TROY*. Results showed *TROY* overexpression dramatically enhanced ALDH activity in HCC cells (Fig. 2E), indicating the cancer stemness maintenance function of *TROY* in HCC.

To further investigate the role of *TROY *in vivo, we established a xenograft mice model via subcutaneous injection of empty vector- and *TROY*-transfected HCC cells into the left and right dorsal flanks of nude mice, respectively. *TROY*-transfected cells were found to overtly increase xenograft tumor growth (Fig. 2F, G, Supplementary Fig. 4D-F).

### Silencing of TROY Impairs cancer stemness in HCC cells

**Fig. 3:**
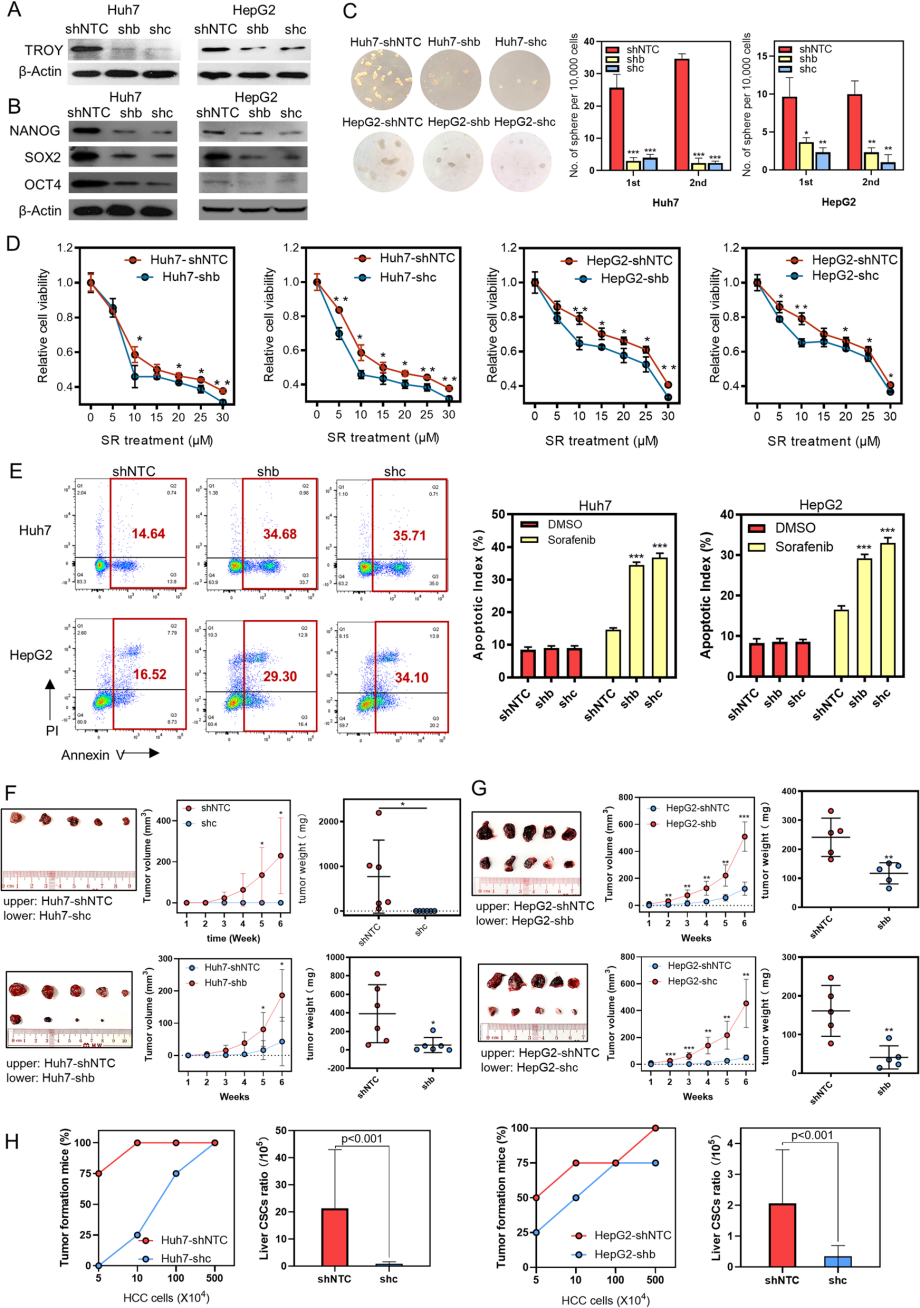
Knockdown of *TROY* inhibits stemness properties of HCC cells. (**A**, **B**) Western blotting of *TROY* (**A**), NANOG, SOX2, and OCT3/4 (**B**) in HepG2 and Huh7 HCC cells transfected with lentiviruses expressing control shRNA (shNTC) or two independent shRNAs targeting *TROY* (shb and shc). β-Actin was used as a loading control. (**C**) Spheroid formation assay (left) and summaries (right) of spheroid numbers in HepG2 and huh7 cells transfected with shNTC or *TROY* shRNAs. (**D**) XTT assay reveals a stronger chemoresistance ability of cells with higher *TROY* expression compared with that of lower *TROY* expression. Cells were treated with the indicated concentration of Sorafenib (SR) for 48 h. **(E**) Representative flow cytometry plots (left) and summary (right) of apoptotic cells in HepG2 and huh7 cells transfected with shNTC or *TROY* shRNAs. Cells were treated with Sorafenib (20 μm) for 24 h. (**F**) In vivo subcutaneous implantation NOD/SCID mice model induced by HepG2 cells transfected with shNTC or *TROY* shRNAs. (**G**) In vivo subcutaneous implantation nude mice model induced by huh7 cells with shNTC or *TROY* shRNAs. Statistical significances: *, *P* < 0.05; **, *P* < 0.01; ***, *P* < 0.001

To confirm whether *TROY* is required for the cancer stemness maintenance of HCC cells, we silenced *TROY* expression with two short hairpin RNAs (shRNA) against *TROY* in HCC cell lines Huh7 and HepG2. The silencing effect was detected by qPCR (Supplementary Fig. 5A), immunofluorescence (Supplementary Fig. 5^1^B), and western blot analysis (Fig. 3A). The results found that *TROY* silencing could decrease the expressions of *NANOG, OCT4,* and *SOX2* by qPCR (Supplementary Fig. 5C), immunofluorescence (Supplementary Fig. 5D), and western blot analysis (Fig. 3B). Next, functional assays were performed in *TROY*-silenced HCC cells, and results showed knockdown of *TROY* dramatically suppressed the abilities of spheroid formation (Fig. 3C), foci formation (Supplementary Fig. 6A), and cell proliferation (Supplementary Fig. 6B). Chemo-drug sensitivity was investigated by XTT and flow cytometry assays. Results demonstrated that *TROY* silencing impaired the cell cycle process (Supplementary Fig. 6C, 6D), the cell viability (Fig. 3D, Supplementary Fig. 6E) and increased the apoptotic index (Fig. 3E) of HCC cells in the presence of sorafenib, cisplatin, and 5-Fu. Furthermore, the in vivo tumor growth assay showed that knockdown of *TROY* significantly decreased the tumor growth (Fig. 3F, G), tumor-initiating capacity, and liver CSCs ratio (Fig. 3H).

### TROY promotes cell migration and metastasis by inducing EMT

**Fig. 4:**
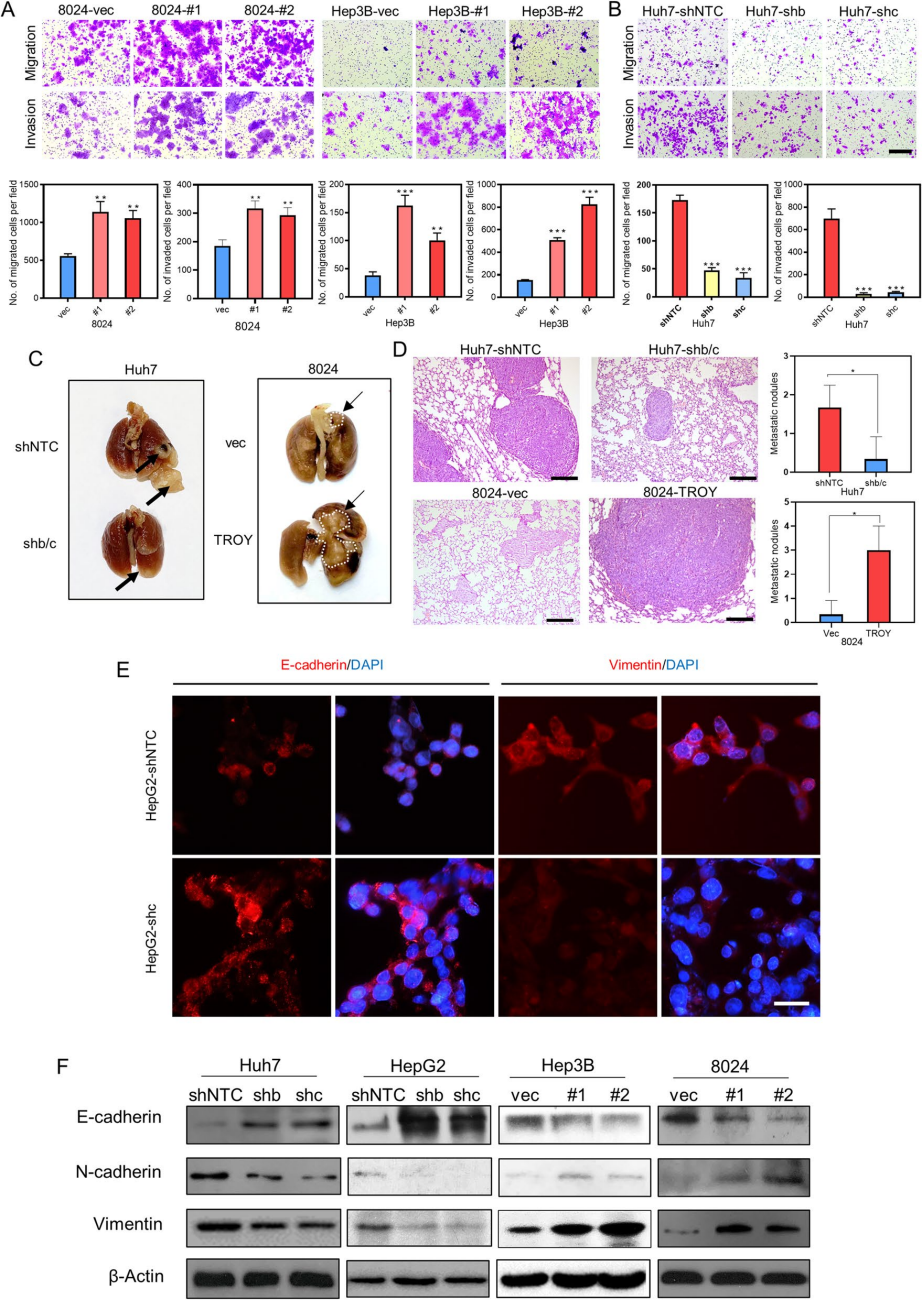
*TROY* promotes HCC migration, invasion and metastasis via EMT. (**A**, **B**) Representative images of transwell migration and matrigel invasion assays (upper) and their summaries (lower) in 8024 or Hep3B cells transfected with vec or *TROY* isoforms (**A**), as well as in Huh7 cells transfected with shNTC or *TROY* shRNAs (**B**). Scale bar = 200 μm. (**C**, **D**) Representative images of lungs (**C**) derived from nude mice after tail vein injection of Huh7 cells transfected with shNTC or *TROY* shRNAs and 8024 cells transfected with vec or *TROY* isoforms and representative H&E staining images (**D**) of the corresponding lung sections. Scale bar = 100 μm. The number of metastatic nodules on the surface (indicated by black arrows) is summarized in the bar charts (*n* = 4). (**E**) Representative immunofluorescence images of E-cadherin and vimentin in HepG2 transduced with shNTC or *TROY* shRNAs. Scale bar = 100 μm. (**F**) Western blotting of E-cadherin, N-cadherin, and vimentin in HCC cells transfected with vec and isoforms, or shNTC and shRNAs. β-Actin was used as a loading control. Statistical significances: *, *P* < 0.05; **, *P* < 0.01; ***, *P* < 0.001

As a high expression of *TROY* was closely associated with metastatic status in HCC patients (Fig. 1H), the effects of *TROY* on cell motility and metastasis were studied by both in vitro and in vivo assays. Cell migration and invasion assays showed that overexpression of *TROY* significantly enhanced HCC cell motility (Fig. 4A, Supplementary 7A). Conversely, the migrative and invasive abilities of HCC cells were impaired when *TROY* was silenced by shRNAs (Fig. 4B, Supplementary 7B). For in vivo lung metastatic mouse model, 10 weeks after tail vein injection of HCC cells, only a few nodules were observed in 1/4 of mice induced by sh*TROY*-transfected Huh7 cells, whereas metastatic nodules on lung surfaces were detected in 4/4 of tested mice injected with shNTC-transfected cells (Fig. 4C). In addition, 4/4 of mice injected with *TROY*-overexpressing PLC8024 cells formed multiple metastatic nodules, however, only 2/4 of mice were found small metastatic nodules on the lung surfaces. H&E staining was used to further confirm the lung metastasis lesions (Fig. 4D). Taken together, these findings strongly suggested that *TROY* could promote HCC metastasis.

To investigate whether this metastatic ability was caused by epithelial-mesenchymal transition (EMT), immunofluorescence staining (Fig. 4E), and western blot (Fig. 4F) analysis was performed to investigate the expression pattern of representative markers. The results revealed that *TROY* could downregulate epithelial marker E-cadherin and increase mesenchymal markers N-cadherin and Vimentin, indicating that *TROY* could promote EMT in HCC cells.

### TROY activates PI3K/AKT/TBX3 axis and upregulates TBX3

**Fig. 5:**
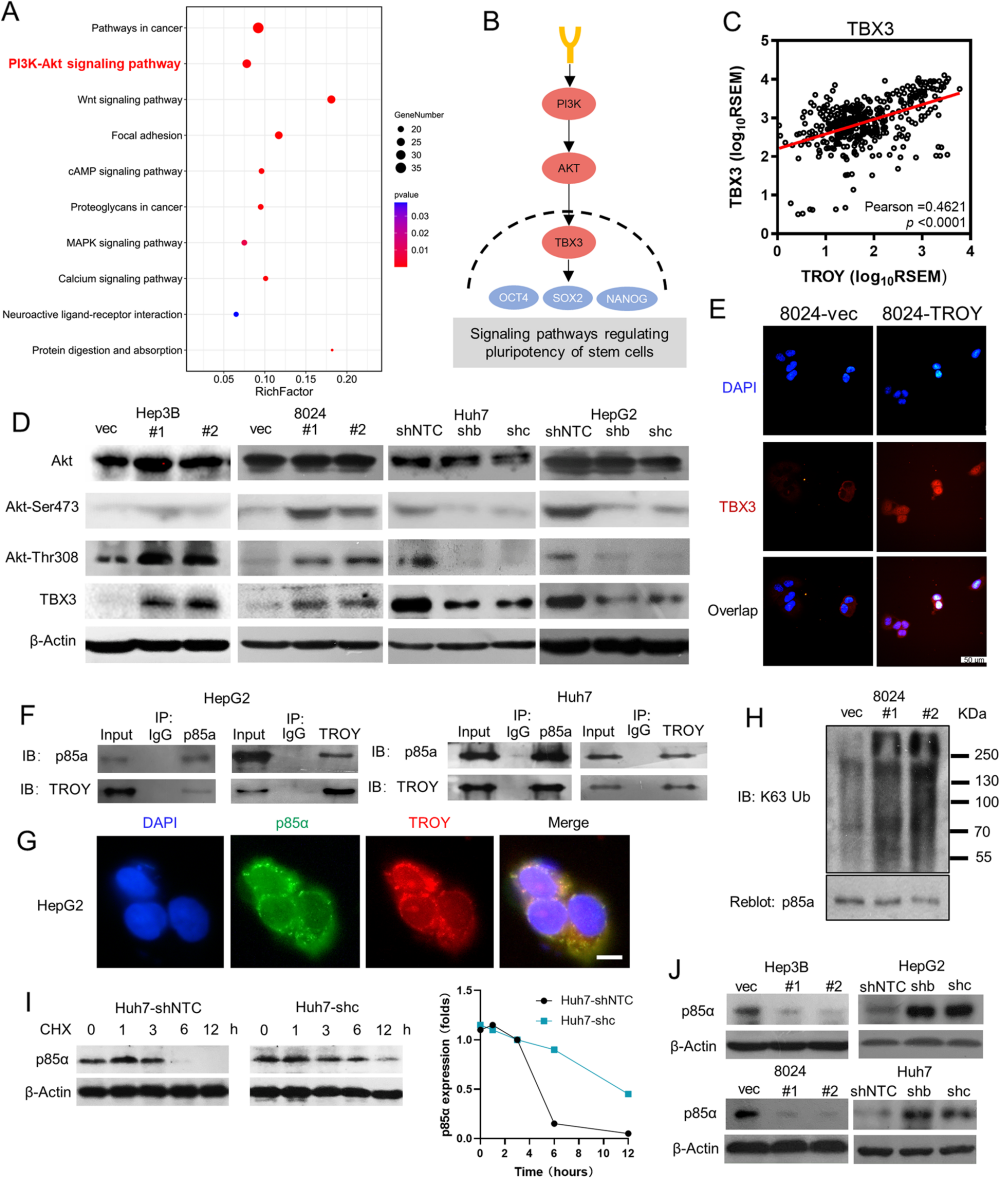
*TROY* activates PI3K/AKT/TBX3 signaling pathway via polyubiquitin p85a. (**A**) KEGG pathway enrichment study of 823 up-regulated genes in *TROY*^hi^ HCC patients. (**B**) PI3K/AKT/TBX3 signaling pathway from Signaling pathways regulating pluripotency of stem cells^21^. (**C**) Co-expression analysis of *TROY* and TBX3 from TCGA datasets. The R-value was detected by Pearson correlation, the *P*-value was tested by independent-samples t-test. (**D**) Western blotting of Akt, Phospho-Akt (Ser473 and Thr308), and TBX3 in *TROY* overexpression and knockdown HCC cells. β-Actin was used as a loading control. (**E**) Representative immunofluorescence images of TBX3 in 8024 transduced with vector or TROY. Scale bar = 50 μm. (**F**) Cell lysates prepared from HepG2 and Huh7 were subjected to immunoprecipitation (IP) with *TROY* antibody or control immunoglobulin G (IgG) and then immunoblotted with p85α antibody. (**G**) IF double staining of *TROY* and p85α in HepG2 cells. Nuclei were stained with DAPI. Scale bar, 20 μm. (**H**) IP with anti-p85α antibody and blotted with anti-k64 antibody in 8024 cells transfected with vec or *TROY* isoforms. (**I**) Western blotting (Left) and quantification (right) of ShNTC- or sh*TROY*-Huh7 cells incubated with CHX (40 μmol/L) for the indicated time points. **(J**) Western blotting of p85α in *TROY* overexpression and knockdown HCC cells. β-Actin was used as a loading control. Statistical significances: ***, *P* < 0.001

To characterize the underlying molecular mechanism of *TROY* in stemness regulation, we analyzed the 823 up-regulated genes in *TROY*^*hi*^ HCC patients. The KEGG pathway result revealed that these genes were enriched in “Pathways in cancer”, “PI3K-AKT signaling” and “Signaling pathways regulating pluripotency of stem cells” (Fig. 5A, Supplementary Table 4). Among these signaling, PI3K/AKT signaling was involved in all three pathways. Furthermore, PI3K/AKT/TBX3 axis which was included in “Signaling pathways regulating pluripotency of stem cells” plays an important role in maintaining pluripotency of mouse ES cells and it has been reported as a treatment target in multi-type of embryonal cancers (Fig. 5B). Gene co-expression analysis using 366 pairs of HCC samples found that the expression of *TROY* was positively correlated with *TBX3* expression (Fig. 5C), suggesting that TROY may be involved in stemness regulation via the control of PI3K/AKT/TBX3 signaling. To validate the result, western blotting (Fig. 5D) and qPCR (Supplementary Fig. 7C) were performed to examine the expression of AKT/TBX3 signaling. As expected, TBX3 expression and phosphorylation of AKT were induced in *TROY*-overexpressing HCC cells, while decreased upon *TROY* silencing. Evidence had already demonstrated that TBX3 regulated stem cell maintenance via controlling stem cell self-renewal and differentiation [24]. It has no known function in adult tissues but is frequently overexpressed in a wide range of epithelial and mesenchymal derived cancers [25]. Interestingly, we found that TBX3 protein is upregulated and translocated from cytoplasm into the nucleus upon TROY overexpression (Fig. 5E), demonstrating that TROY’s overexpression activated the transcriptional activator function of TBX3 in the nucleus. Collectively, these results demonstrated that *TROY* activates PI3K/AKT signaling and upregulates TBX3 expression in HCC. To further validate the role of TBX3 in TROY-activated PI3K/AKT pathway, we knockout of *TBX3* in *TROY* overexpression HCC cell lines. Western blotting results revealed that expressions of NANOG, SOX2, and OCT4 were dramatically downregulated upon TBX3 knockout in *TROY* overexpression HCC cell lines (Supplementary Fig. 7D). Functional studies showed that drug resistance (Supplementary Fig. 7E) and spheroid formation ability (Supplementary Fig. 7F) were decreased upon TBX3 knockout in TROY overexpression HCC cells.

### TROY interacts with P85α and induces its polyubiquitylation

Next, we investigated the molecular mechanism of how *TROY* activates the PI3K/AKT/TBX3 signaling. By examining the interactive proteins of TROY via the online protein–protein interaction database [26], we found that Phosphoinositide-3-Kinase Regulatory Subunit 1 (PIK3R1, also named p85α), a predominant regulatory subunit of PI3K has been reported to be able to bind TROY (Supplementary Fig. 8A). Immunoprecipitation analysis was then conducted to confirm the interaction between TROY and p85α, and the result showed that p85α was bound with TROY in both HepG2 and Huh7 cells (Fig. 5F). Immunofluorescent staining further confirmed the colocalization of TROY and p85α in HCC cells (Fig. 5G). An important mechanism of the regulation of signaling pathways was posttranscriptional modifications such as phosphorylation or ubiquitylation. We then asked whether the interaction of TROY with p85α was able to induce p85α phosphorylation or ubiquitylation. GSEA analysis result showed that the GO term “K63 linked polyubiquitin modification dependent protein binding” was skewed toward the *TROY*^hi^ group (Supplementary Fig. 8B). The western blotting results demonstrated that the Lys63-linked polyubiquitylation was highly upregulated in *TROY*-overexpressing PLC8024 cells, accompanied by p85α protein degradation (Fig. 5H). Knockdown of *TROY* could effectively inhibit the degradation rate of p85α in cycloheximide (CHX)-treated Huh7 cells, and markedly decrease the half-life of p85α degradation from 3 to 6 h (Fig. 5I). Consistently, western blot results showed the expression of p85α was evidently decreased upon *TROY* overexpression in HepG2 and PLC8024 cells, while increased when silencing of *TROY* in Huh7 and Hep3B cells (Fig. 5J), suggesting that TROY plays an important role in p85α degradation via polyubiquitylation.

### Loss of P85α is required for PI3K-AKT Activation and TROY-mediated cancer stemness maintenance

**Fig. 6:**
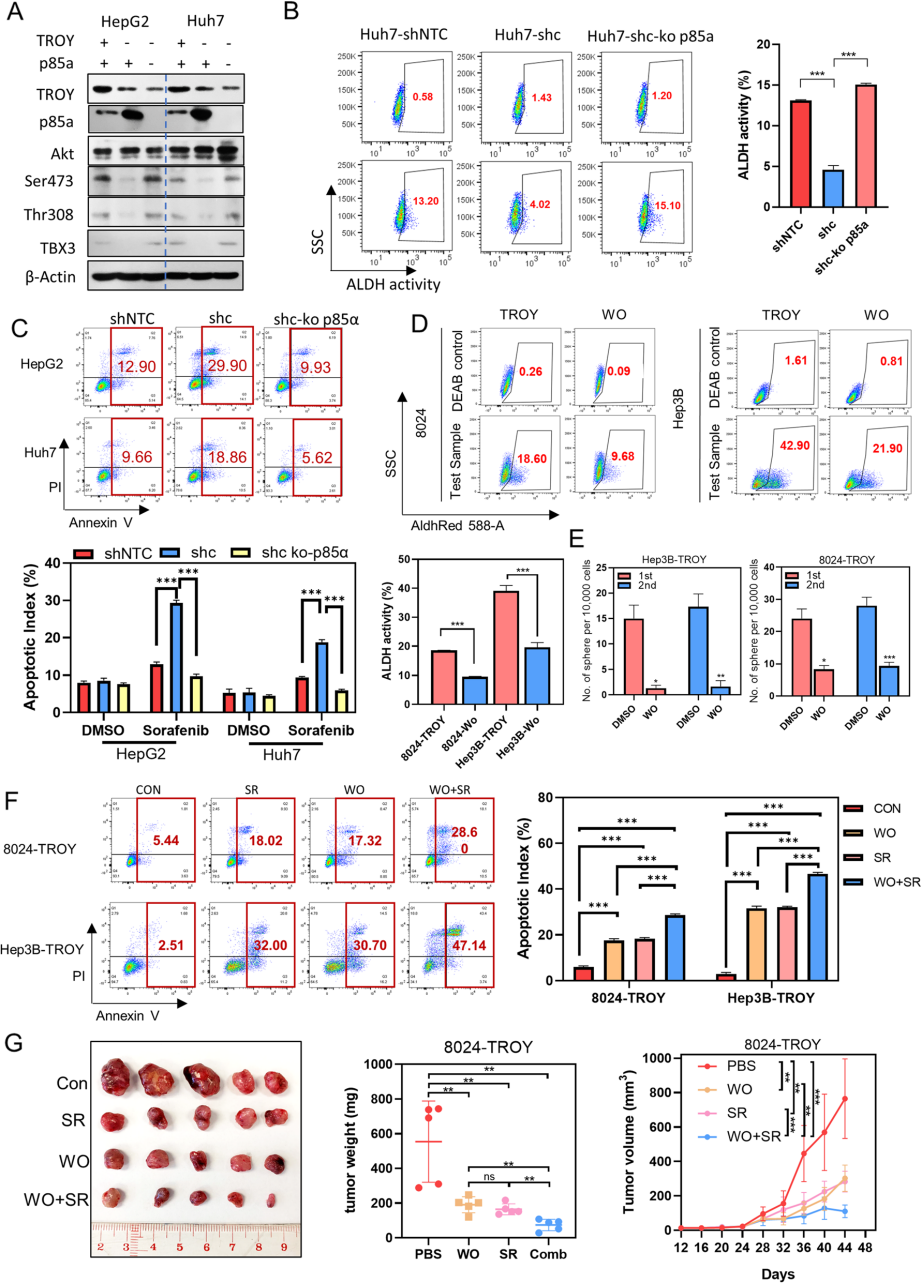
Downregulation of p85a or inhibition of PI3K/AKT pathway can abolish the oncogenic effects of *TROY*. (**A**) Western blotting of p85α, Akt, Phospho-Akt (Ser473 and Thr308), and TBX3 in Huh7 and HepG2 cells transfected with *TROY*-shNTC or *TROY*-shc or *TROY*-shc knockout p85α. (**B**) Representative flow cytometry plots (left) and summaries (right) of ALDH activity in Huh7 cells transfected with *TROY*-shNTC or *TROY*-shc or *TROY*-shc knockout p85α. (**C**) Representative flow cytometry plots (upper) and summaries (lower) of a percentage of apoptotic cells in Huh7 and Hepg2 cells transfected with *TROY*-shNTC or *TROY*-shc or *TROY*-shc knockout p85α. Cells were treated with Sorafenib (20 μm) for 24 h. (**D**) Representative flow cytometry plots (upper) and summaries (lower) of ALDH activity in 8024-*TROY* and Hep3B-*TROY* cells treated with DMSO or Wortmannin (50 nM). (**E**) Summaries of spheroid numbers of 8024-*TROY* and Hep3B-*TROY* cells treated with DMSO or Wortmannin (WO) (50 nM). (**F**) Representative flow cytometry plots (left) and summaries (right) of a percentage of apoptotic cells in 8024-*TROY* and Hep3B-*TROY* cells treated with DMSO, sorafenib (20 μm), Wortmannin (50 nM) or the combination of sorafenib (20 μm) and Wortmannin (50 nM). (**G**) Mice treated with a combination of sorafenib and Wortmannin significantly reduced the tumor growth rate when compared with a single treatment

Given that p85α has been well characterized to negatively regulate PI3K pathway [27], we explored whether loss of p85α could lead to PI3K-AKT activation. *CRISPR-Cas9* was applied to knock out *p85α* expression in *TROY*-silenced HCC cells. Western blotting results revealed that expressions of p-AKT and TBX3 were re-activated upon *p85α* knockout (Fig. 6A), indicating p85α acting upstream of PI3K. Functional studies including ALDH, apoptosis, and sphere formation assays were performed to evaluate the role of p85α in *TROY*-mediated cancer stemness maintenance. The results showed that the ALDH activity (Fig. 6B) and drug resistance (Fig. 6C) were regained upon *p85α* knockout in *TROY*-silenced HCC cells.

To further explore the role of PI3K-AKT signaling in *TROY*-mediated cancer stemness maintenance, PI3K inhibitor wortmannin was applied in *TROY*-expressing HCC cells. The result showed that wortmannin treatment abrogates *TROY*-induced ALDH activity (Fig. 6D) and spheroid formation (Fig. 6E, Supplementary Fig. 8C).

In the clinic, sorafenib, an FDA-approved tyrosine kinase inhibitor for the first-line therapy of advanced HCC patients, significantly against protein kinases including VEGFR, PDGFR, and RAF kinases [28]. Increasing evidence has demonstrated that drug resistance of sorafenib can be acquired by cancer cells by activating signaling pathways including PI3K/AKT signaling [29]; nevertheless, the detailed mechanism for the activation is not fully understood. Here, we treated *TROY*-expressing HCC cells with both sorafenib and wortmannin. Interestingly, the apoptosis experiment result showed that the combination of wortmannin and sorafenib can enhance the inhibitory of HCC cell lines (Fig. 6F). Furthermore, mice with tumors induced by *TROY*-expressed HCC cells were given either vehicle control, wortmannin, sorafenib, or a combination of both for 16 days. Compared with the vehicle control group, wortmannin, sorafenib and combined treatment groups all demonstrated reduced tumor size and weight, and the combined treatment group showed the maximal suppression of tumors (Fig. 6G). The bodyweight of mice was measured as an indication of drug toxicity, and no significant difference among the four treatment groups was observed (Supplementary Fig. 8D).

### CAF-derived TGF-β1 upregulates TROY and activates PI3K/AKT/TBX3 signaling

**Fig. 7:**
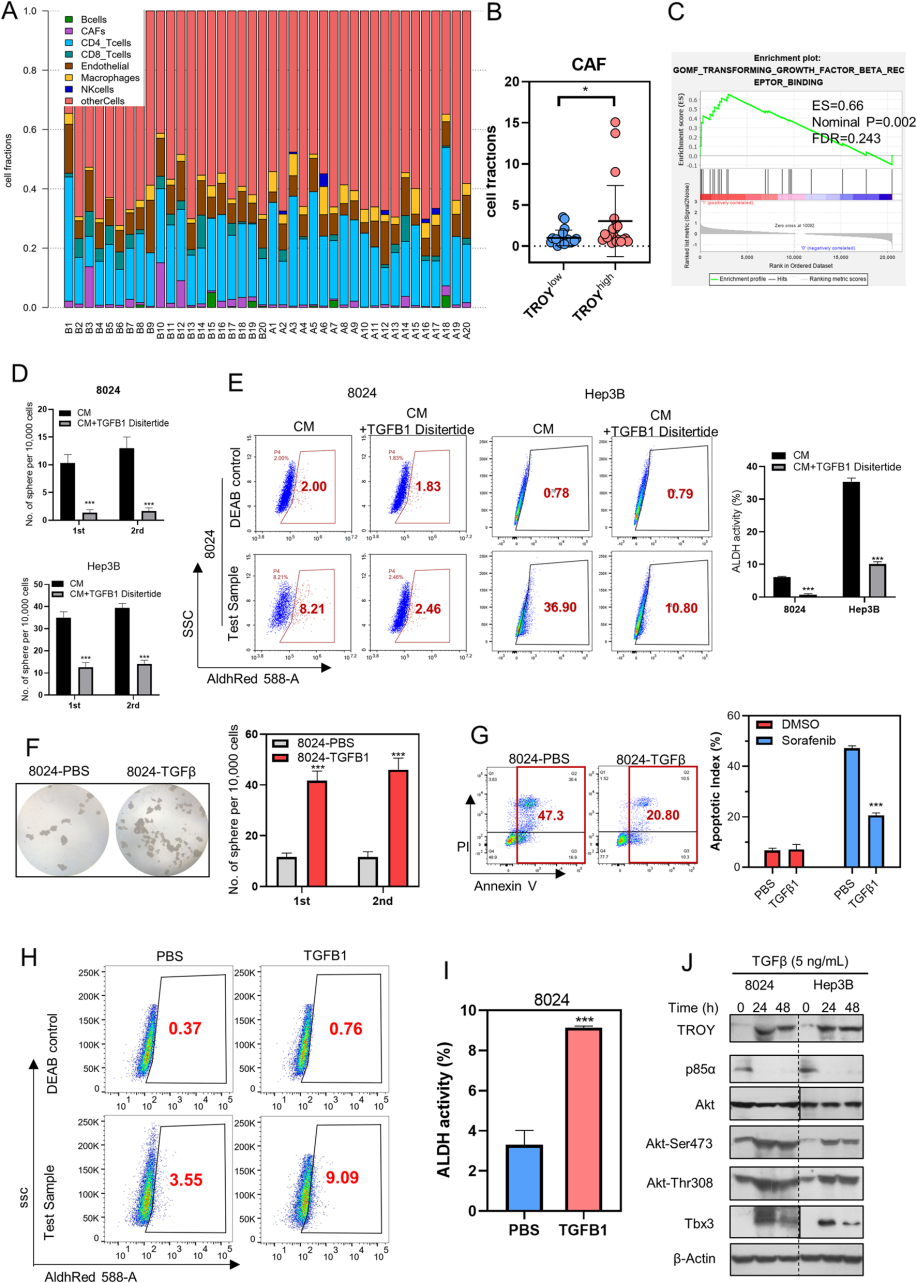
CAF-derived TGF-β1 enhances the cancer stemness via upregulating *TROY*. (**A**) Percentage of immune cells in *TROY*^hi^ expression patients (B1-B20) and *TROY*^low^ expression patients (A1-A20) from TCGA database. (**B**) Fraction of CAF cells in TROY^hi^ and TROY^low^ patients. (**C**) GSEA analysis of TROY^high^ groups was enriched in TGFβ1 receptor binding. (**D**) Spheroid formation assay (left) and the spheroid numbers (right) in 8024 cells treated with PBS or TGFβ1 (5 ng/ml, 24 h). (**E**) Representative flow cytometry plots (left) and summaries (right) of a percentage of apoptotic cells in 8024 cells treated with PBS or TGFβ1 (5 ng/ml, 24 h). Cells were treated with Sorafenib (20 μm) for 24 h. (**F**) Representative flow cytometry plots (left) and summaries (right) of ALDH activity in 8024 treated with PBS or TGFβ1(24 h). (**G**) Western blotting of *TROY*, p85α, Akt, Phospho-Akt (Ser473 and Thr308) and TBX3 in 8024 and Hep3B cells treated with PBS (0 h) or TGFβ1 (5 ng/ml, 24 h, and 48 h). (**H**) Summary of pathological and underlying regulatory mechanisms of *TROY* complex in hepatocellular carcinoma

To explore the mechanism of *TROY* upregulation in HCC, we used EPIC [30] (http://epic.gfellerlab.org) to predict the tumor microenvironment in *TROY*^hi^ and *TROY*^lo^ patient groups. Interestingly, the number of cancer associate fibroblasts (CAFs) was found significantly higher in *TROY*^hi^ patient samples (Fig. 7A and B). GSEA results showed that high expression of TROY is significantly associated with the established gene sets “TGF-β receptor binding” (Fig. 7C). TGF-β1, mainly secreted by CAF, has been well characterized as a messenger between tumors and fibroblasts and played a significant role in tumor migration and stemnesss [31, 32]. IHC staining confirmed that TGF-β was secreted by CAFs rather than other cells in HCC specimens (Supplementary Fig. 8E). Hence, we hypothesized that CAF-derived TGF-β1 upregulated TROY expression in tumor cells. Disitertide, a TGF-β1 inhibitor, reduced the spheroid formation ability (Fig. 7D, Supplementary Fig. 8F) and ALDH expression (Fig. 7E) in HCC cell lines co-cultured with CAF conditional medium (CM). Consequently, depletion of TGF-β1 abolished the stemness induced by CAF conditional medium. Next, functional assays were conducted to assess the effect of TGF-β1 on cancer stemness maintenance. Indeed, the results showed that TGF-β1 treatment promoted both primary and secondary spheroid formation ability (Fig. 7F), drug resistance ability (Fig. 7G, Supplementary Fig. 8G), and the ALDH activity (Fig. 7H) in HCC cells. Western Blot results showed that TGF-β1 treatment could effectively upregulate TROY expression and activate PI3K/AKT/TBX3 signaling in HCC cells (Fig. 7I, Supplementary Fig. 8H). Collectively, our findings suggested that CAF-derived TGF-β1 upregulates TROY and activates PI3K/AKT/TBX3 signaling in HCC cell lines.

## Discussion

The existence of CSCs is one of the major contributors of malignant phenotypes of HCC such as high tumor recurrence, metastasis, and resistance to conventional chemotherapy or radiotherapy. Therefore, the development of therapeutic strategies targeting CSCs will be promising in HCC treatment. Emerging evidence suggests that CSCs share similar phenotypes and regulatory networks with normal tissue progenitor cells. In addition, poor-differentiated HCCs always demonstrated lineage-reversed phenotype with unsatisfactory prognosis [33]. To explore key signaling involved in the CSC regulation in HCC, we established an in vitro hepatocyte differentiation model, which was induced from ES cells to mimic liver development and focused on genes specifically expressed in liver progenitors. Among them, *TROY* was specifically expressed in the liver progenitor stage, shut downed in mature hepatocytes, and re-activated in HCC.

TROY belongs to the TNF receptor superfamily consisting of 19 TNFRSF ligands and 29 different TNFRSF receptor [12]. Interactions between the TNFRSF ligands and TNFRSF receptors activate downstream signaling and regulate multiple biological processes such as cell survival, proliferation, and differentiation [34]. However, the ligand of TROY has not been found yet. Previous studies found that TROY does not bind to any known TNF ligands, and its intracellular domain is distinct from other characterized members of the TNF receptor superfamily. In the present study, we firstly reported that TROY could interact with p85α and play an important role in its k63 ubiquitination and degradation. The molecular mechanism of how TROY induced k63 ubiquitination was not explored in the present work. However, we found that TROY could recruit TRAF family members (TRAF1, TRAF2, TRAF3, and TRAF5) and E3 ubiquitin-protein ligases (ITCH, NEDD4, NEDD4L, WWP1, and FBXW7) through direct protein–protein interaction (Supplementary Fig. 5C), suggesting its role in regulating k63 ubiquitination of p85α.

Previous studies reported that TROY is a marker of normal tissue stem cells in adult kidney [14], brain [11], and stomach [15]. In mouse intestinal tissue, TROY was found to interact with similar signaling pathways activated in ESs or cell lineage differentiation [13]. In the context of cancers, TROY was found to promote tumor progression via multiple signaling pathways. For example, TROY exerted its oncogenic function in glioma through activating nuclear factor kappa B (NF-κB) signaling [17] and increased tumorigenesis in nasopharyngeal carcinoma by suppressing TGFβ signaling [18]. In the present study, we found that TROY-expressing cells could not be detected in normal liver tissues, while a small number of TROY-expressing cells could be observed in HCC cells, which is consistent with the characteristic of CSCs. Interestingly, the high-frequency of TROY-expressing cells was negatively associated with poor HCC outcome (*P* = 0.02) as well as tumor metastasis.

To explore the oncogenic role of TROY in HCC development and progression, both in vitro and in vivo functional assays were applied in *TROY*-expressing cells and *TROY*-silencing cells. The results found that *TROY* had a strong oncogenic ability by promoting cell growth in vitro and tumor formation in vivo. The effect of TROY on stemness regulation was also addressed in the present study, and experimental data suggested that TROY played important role in stemness regulation. Overexpression of *TROY* could increase the abilities of sphere formation and chemoresistance, which could be effectively abrogated in *TROY*- silencing cells. In addition, overexpression of *TROY* could upregulate stemness-related genes such as *SOX2* and *OCT4* in HCC cells. Taken together, these findings revealed that *TROY* plays key roles in HCC progression including metastasis through upregulating HCC stemness.

Next, we characterized the molecular mechanism of TROY in stemness regulation in HCC as a liver CSC marker and found TROY could promote the stemness by activating p85a/AKT/TBX3 pathway. Importantly, p85α acted as a tumor brake in different types of cancers, and the mutations in the i-SH2 region of *p85α* have been frequently detected in tumor tissues, blocking p110α activity and thus activating PI3K/AKT signaling [35]. Interestingly, the deletion of p85α in the mouse liver could lead to gradual morphological changes of hepatocytes and result in the development of HCC [36]. These observations indicated the essential role of *p85α* in suppressing cancer progression. In the present study, the IHC staining demonstrated a decreased expression of the p85α in HCC clinical samples compared with para-tumor tissues. The *TROY* overexpression in HCC cells could downregulate p85 expression and conversely, the silencing of *TROY* upregulated p85α expression, indicating that the *TROY*-mediated p85α downregulation was essential for HCC development and progression.

As a subunit of PI3K, the loss of p85α function contributed to the activation of downstream PI3K/AKT/TBX3 signaling in CSCs. Targeting the activated *TROY*/ p85α/TBX3 axis using the small-molecule PI3K inhibitor wortmannin impairs the tumor stemness properties of HCC cells. Notably, the application of wortmannin increased the sensitivity of HCC cells to sorafenib in both in vitro and in vivo experiments, suggesting that wortmannin might be used as a novel therapeutic strategy to augment the efficacy of sorafenib by targeting liver CSCs.

The crosstalk between tumor cells and stromal cells in the tumor microenvironment has been widely studied and played important roles in tumor progression [37]. Among all the stromal cells that constitute the tumor microenvironment, CAFs were regarded as the most abundant populations that have a great impact on tumor behavior [38]. CAFs exerted their influence on tumor or other stromal cells via direct cell–cell contact or releasing regulatory factors that remodel the microenvironment, such as TGFβ, IL-6, and PDGFs [39]. Importantly, TGFβ is a well-characterized immunosuppressive cytokine mainly secreted by CAFs that is responsible for transforming innate or adaptive immune cells into immunosuppressive cells [40]. In the present study, we found that CAFs secreted TGFβ could increase TROY expression of cancer cells and activate the TROY-mediated PI3K/AKT/TBX3 signaling pathway, promoting stemness properties of HCCs. These findings uncovered the undescribed role of TGFβ in enhancing liver CSCs features and highlighted the importance of targeting TGFβ-induced TROY upregulation and subsequent PI3K/AKT/TBX3 activation.

## Conclusions

Collectively, we defined the role of TROY as a liver CSCs marker that regulates the self-renewal, drug resistance, tumorigenicity, and metastasis of HCC cells. The expression of TROY could be upregulated in HCC tissues by CAFs-derived TGF-β1. Furthermore, we clarified that the interaction between TROY and p85α could lead to p85α degradation and subsequently activate PI3K/AKT/TBX3 signaling, which helps to maintain the pluripotency of liver CSCs by upregulating the expression of SOX2, NANOG, and OCT4 and promote cell motility via activating EMT pathway in HCC. Targeting liver CSCs with PI3K inhibitor wortmannin combined with sorafenib might be a novel therapeutic strategy for HCC patients.

## Supplementary Information

**Additional file 1.**

## Data Availability

This study did not generate any data sets. Source data are provided with this paper.

## Abbreviations

AFP: Alpha-fetoprotein
ALDH: Aldehyde dehydrogenase
CAFs: Cancer-associated fibroblasts
CK19: Cytokeratin 19
CK7: Cytokeratin 7
CSCs: Cancer stem cells
DE: Definitive endoderm
DFS: Disease-free survival
EMT: Epithelial-mesenchymal transition
ES: Embryonic stem cells
GO: Gene Ontology
GSEA: Gene set enrichment analysis
IHC: Immunohistochemistry
IP: Immunoprecipitation
LP: Liver progenitor
NANOG: Nanog homeobox
OCT4: Pou class 5 homeobox 1
P85α: Phosphoinositide-3-kinase regulatory subunit 1
PH: Premature hepatocytes
qPCR: Quantitative real-time PCR
shRNA: Short hairpin RNAs
SOX2: Sry-box transcription factor 2
SOX9: SRY-Box Transcription Factor 9
TBX3: T-box transcription factor 3
TCGA: The cancer genome atlas
TGF-β1: Transforming growth factor beta1
TMA: Tissue microarray
TROY: TNF receptor superfamily member 19
