# Supplementary material for: Targeting TROY-mediated P85a/AKT/TBX3 signaling attenuates tumor stemness and elevates treatment response in hepatocellular carcinoma
Source: J Exp Clin Cancer Res. 2022 May 25;41:182. doi: 10.1186/s13046-022-02401-6 (PMC9131684; doi:10.1186/s13046-022-02401-6)
Supplement: Supplementary file 1 — Additional file 1. [file 13046_2022_2401_MOESM1_ESM.docx]

**SUPPLEMENTARY INFORMATION**

**Targeting *TROY*-mediated P85a/AKT/TBX3 Signaling Attenuates Tumor Stemness and Elevates Treatment Response in Hepatocellular Carcinoma**

**Supplementary Figure 1.**

**
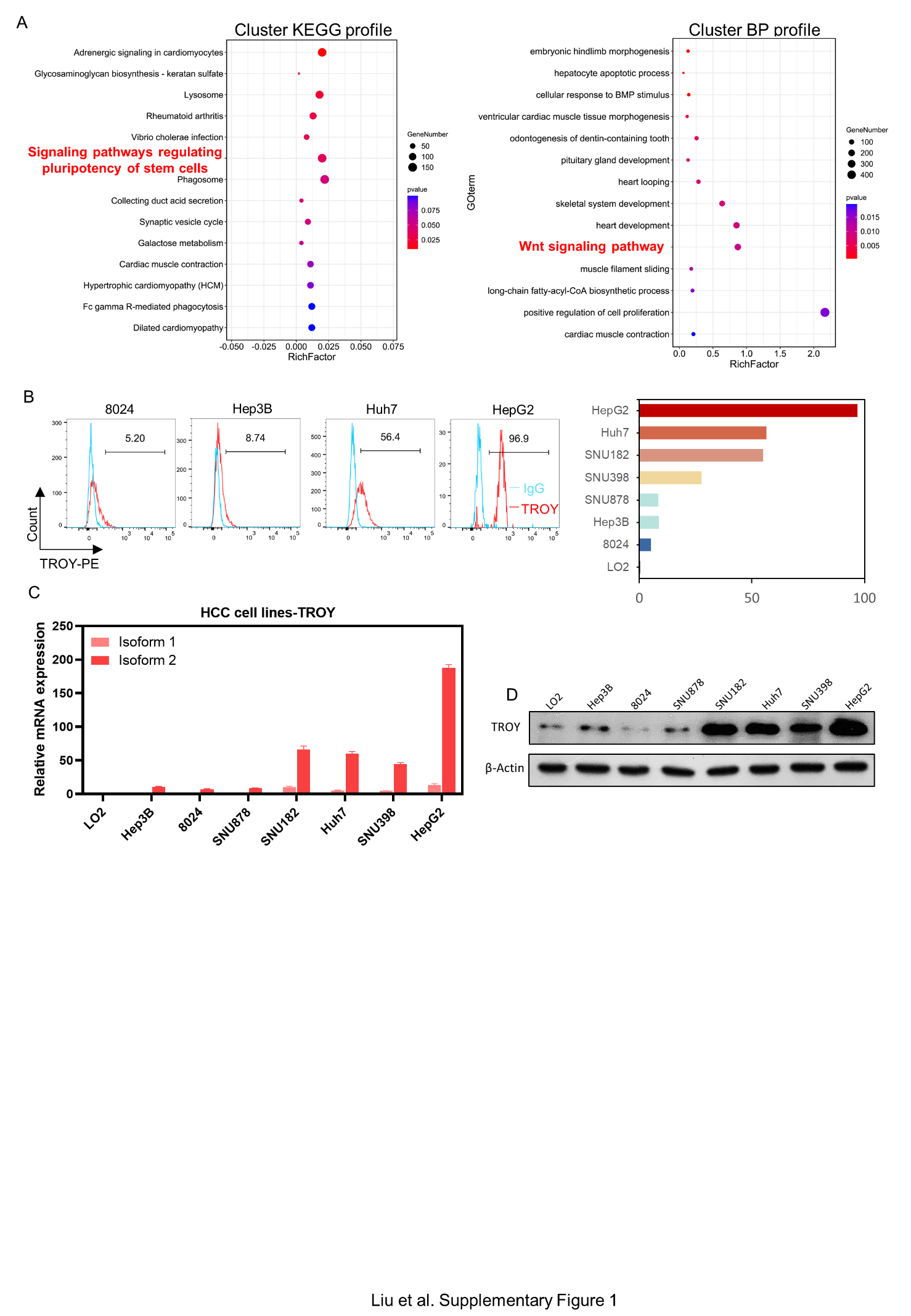
**

(A) KEGG pathway and Biological processes (BP) enrichment analysis of the cluster a cluster of genes with specific expression in liver progenitors.

(B-D) Flow cytometry (B), qPCR (C), and western blot (D) of TROY in HCC cell lines.

**Supplementary Figure 2.**


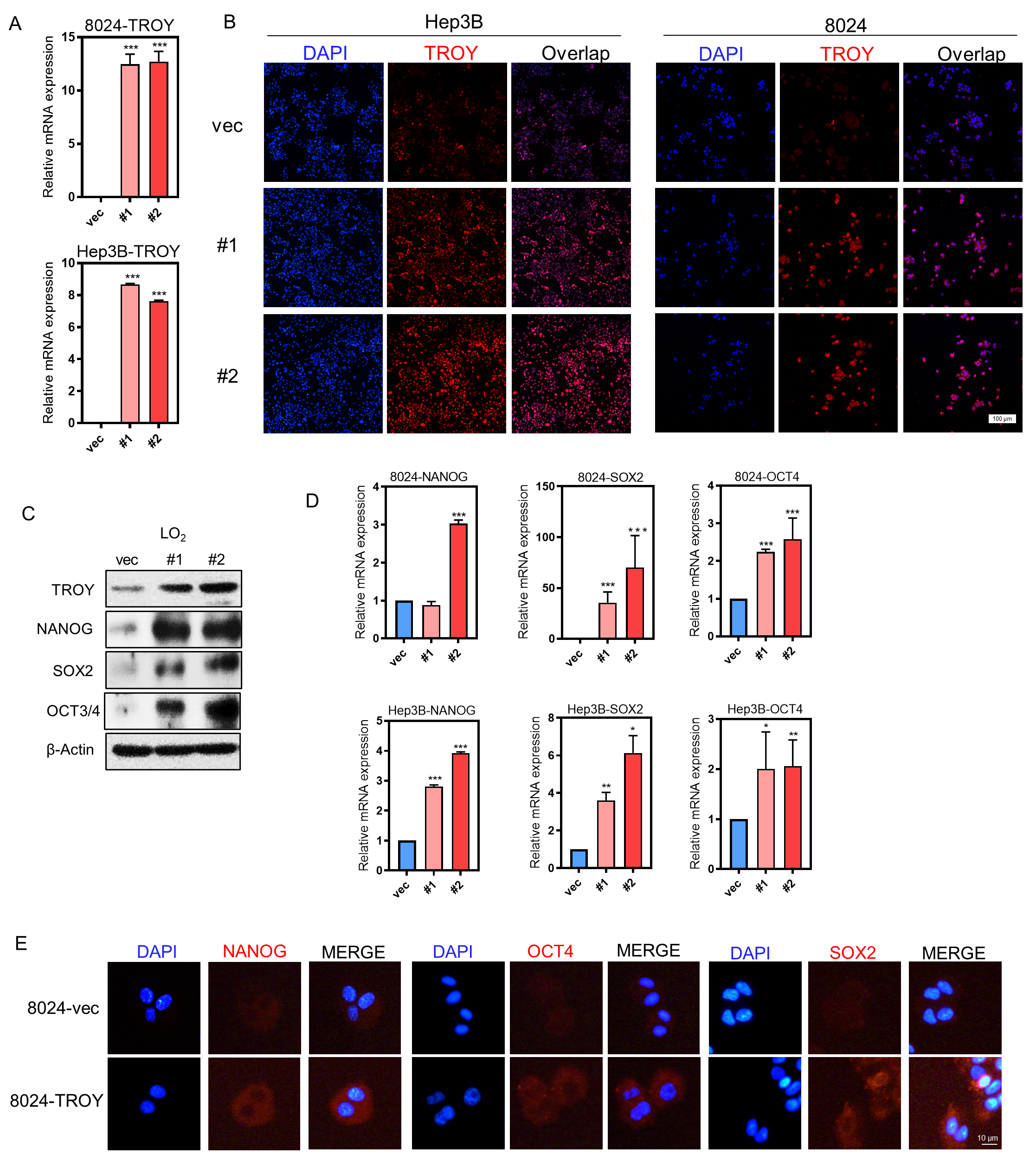


(A) qPCR confirmed the overexpression of TROY in 8024 and Hep3B.

(B) Representative immunofluorescence images of TROY in 8024 and Hep3B cells transfected with vec or TROY isoforms. Scale bar = 100μm.

(C) Western blot of TROY, NANOG, SOX2, OCT3/4 in LO2 with vec or TROY isoforms. β-Actin was used as a loading control in western blot.

(D) qPCR of *NANOG, SOX2, OCT4* in 8024 and Hep3B cells with vec or TROY isoforms.

(E) Representative immunofluorescence images of NANOG, SOX2, OCT4 in 8024 cells transfected with vec or TROY isoforms. Scale bar = 10μm.

**Supplementary Figure 3.**


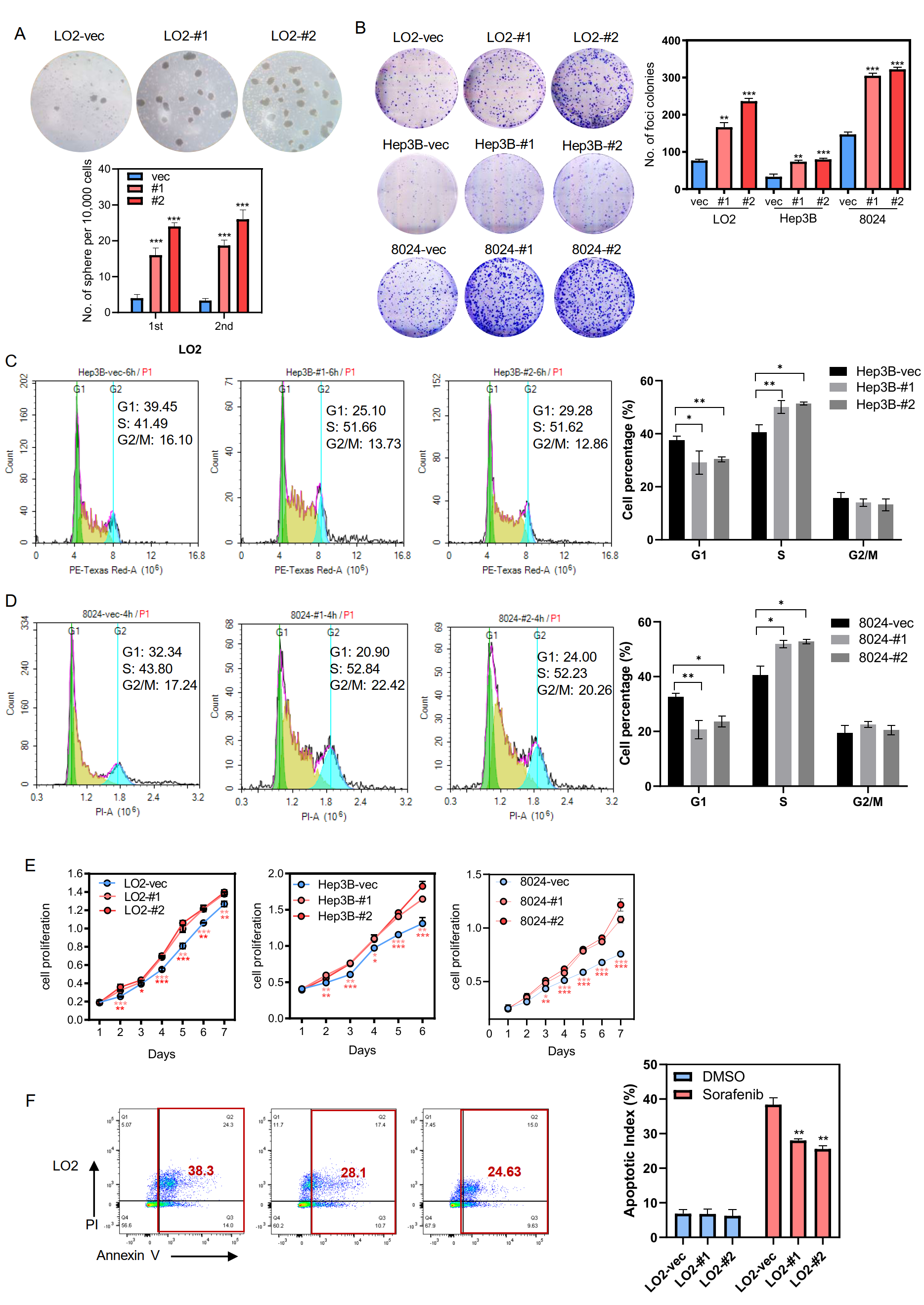


(A) Spheroid formation assay (upper) and statistics(lower) of spheroid numbers in LO2 with vec or TROY isoforms.

(B) Representative foci formation plots (left) and statistics (right) of the number of foci colonies in LO2, 8024, and Hep3B cells with vec or TROY isoforms.

(C) Analysis of cell distribution in each stage of the cell cycle in each transfected Hep3B HCC cells.

(D) Analysis of cell distribution in each stage of the cell cycle in each transfected 8024 HCC cells.

(E) XTT assay was used to determine the cell proliferation rates in LO2, 8024 and Hep3B cells with vec or TROY isoforms.

(F) Representative flow cytometry plots (left) and statistics (right) of the percentage of apoptotic cells in LO2 cells with vec or TROY isoforms.

Statistical significances: *, *P* < 0.05; **, *P* < 0.01; ***, *P* < 0.001.

**Supplementary figure 4.**

**
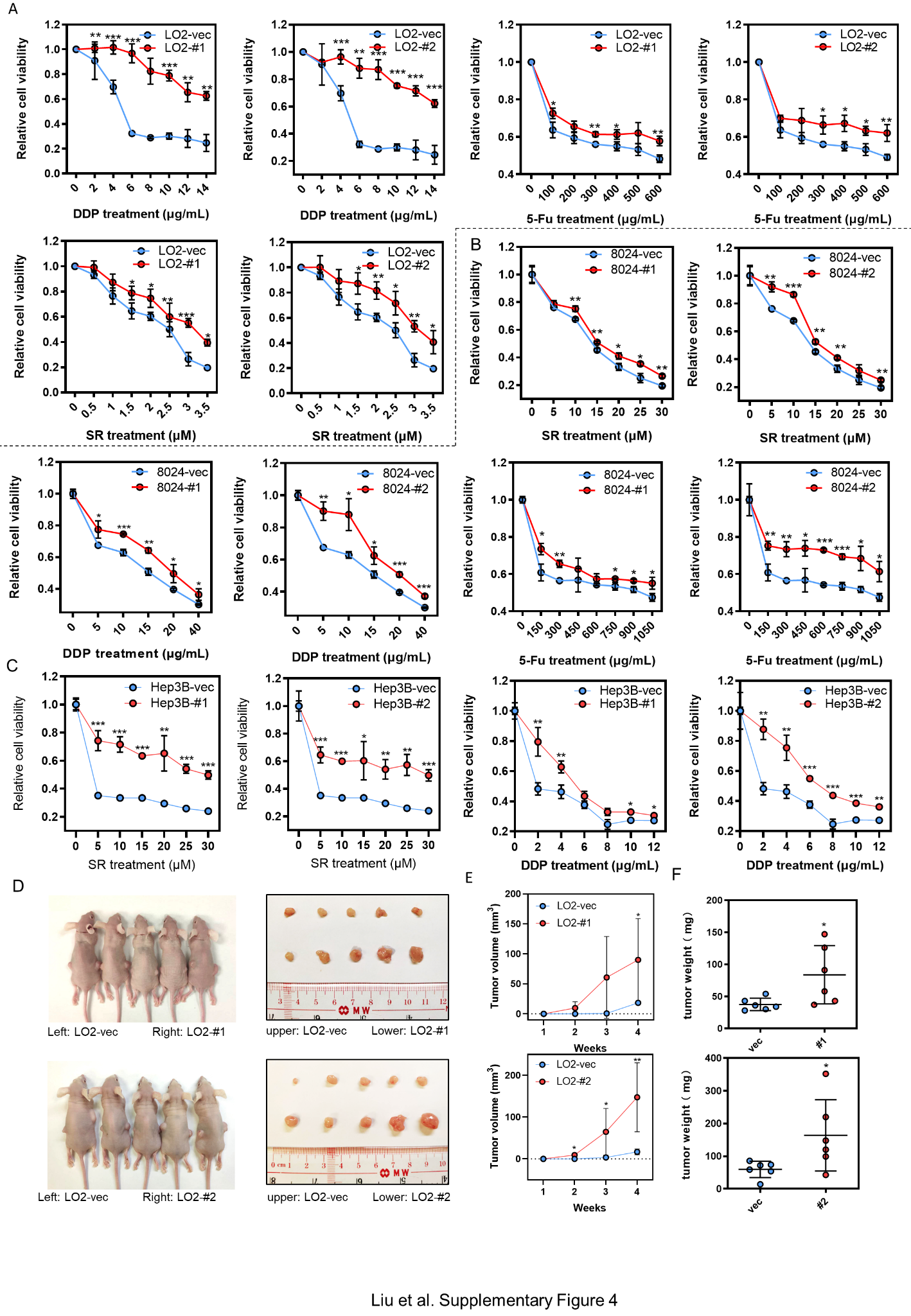
**

1. XTT assay reveals stronger chemoresistance ability of cells with higher TROY expression compared with that of lower TROY expression in LO2. Cells were treated with the indicated concentration of Sorafenib (24h), Cisplatin (DDP) (48 h), or 5-Fu (24h).
2. XTT assay reveals stronger chemoresistance ability of cells with higher TROY expression compared with that of lower TROY expression in 8024. Cells were treated with the indicated concentration of Sorafenib (24h), Cisplatin (DDP) (24 h), or 5-Fu (24h).
3. XTT assay reveals stronger chemoresistance ability of cells with higher TROY expression compared with that of lower TROY expression in Hep3B. Cells were treated with the indicated concentration of Sorafenib (48h), Cisplatin (DDP) (48 h).

(D-F) In vivo subcutaneous implantation nude mice model (D), tumor volume (E), and tumor weight (F) in LO2 cells with vec or TROY isoforms.

Statistical significances: *, *P* < 0.05; **, *P* < 0.01; ***, *P* < 0.001.

**Supplementary Figure 5**


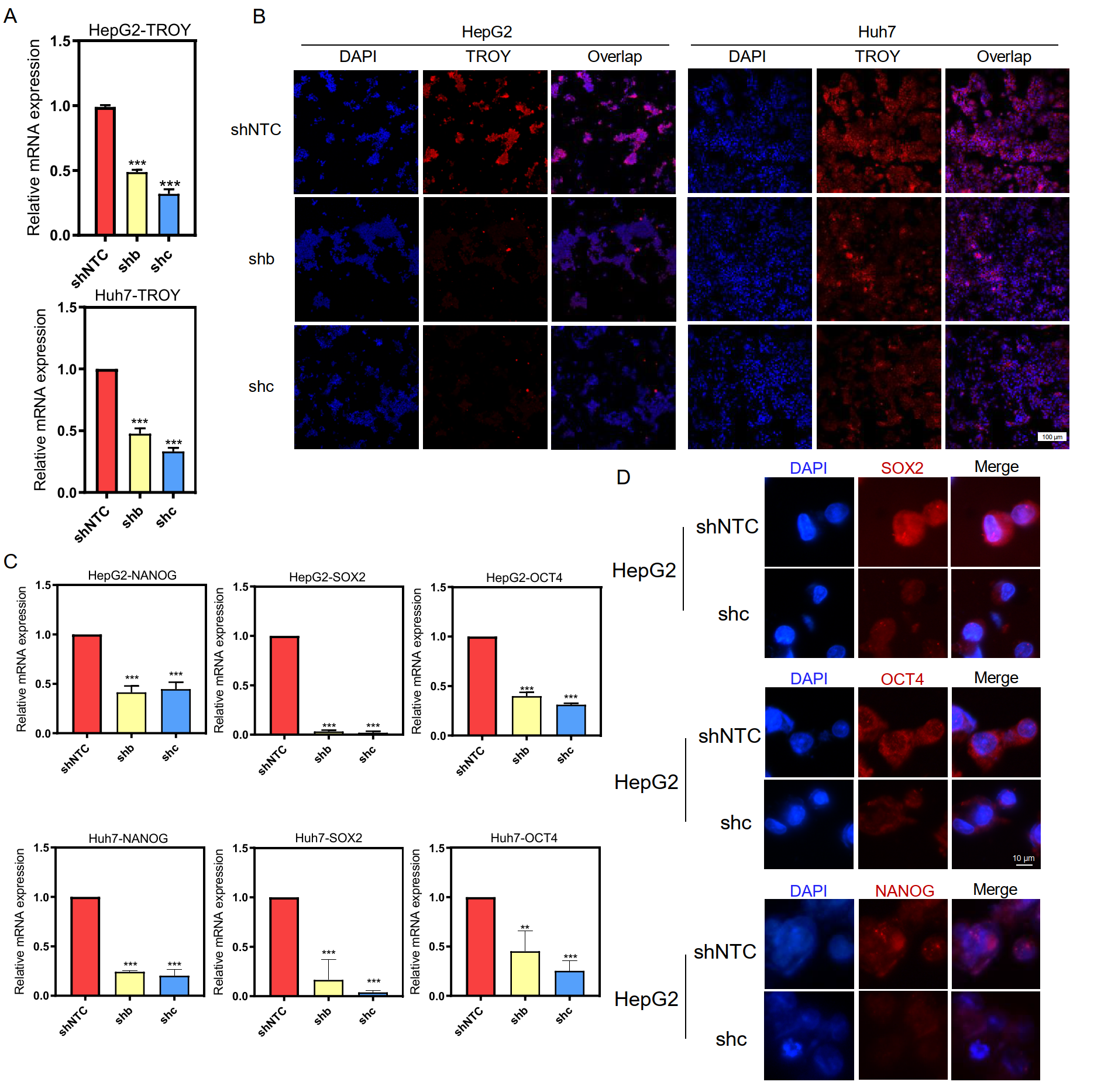


(A) qPCR of *TROY* in HepG2 and huh7 cells with shNTC or TROY shRNAs.

(B) Representative immunofluorescence images of TROY in HepG2 and huh7 cells with shNTC or TROY shRNAs. Scale bar = 100μm.

(C) qPCR of *NANOG, SOX2, OCT4* in HepG2 and huh7 cells with shNTC or TROY shRNAs.

(D) Representative immunofluorescence images of NANOG, SOX2, OCT4 in HepG2 cells with shNTC or TROY shRNA. Scale bar = 10μm.

Statistical significances: *, P < 0.05; **, P < 0.01; ***, P < 0.001.

**Supplementary Figure 6**


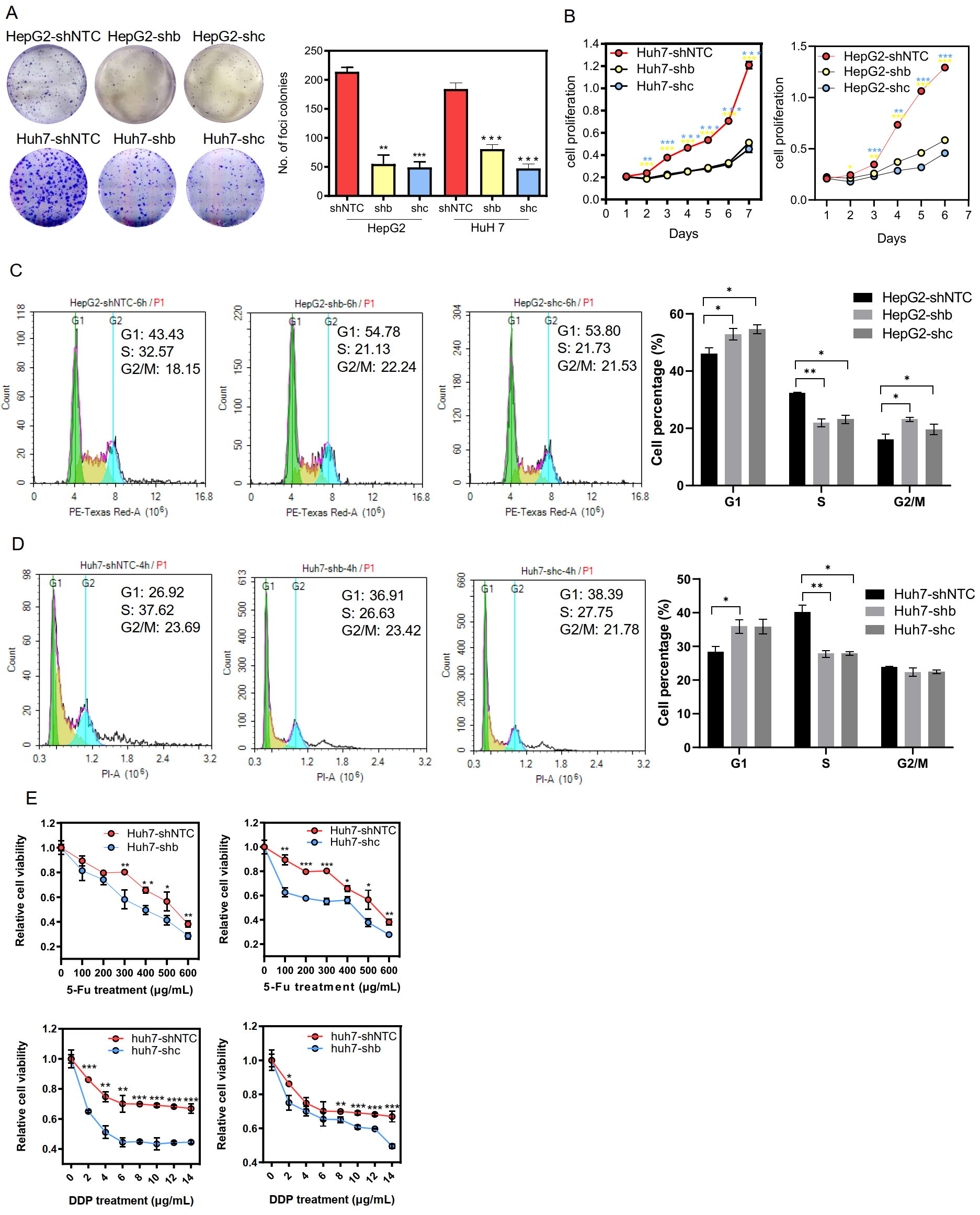


(A) Representative foci formation plots (upper) and statistics (down) of the number of foci colonies in HepG2 and huh7 cells with shNTC or TROY shRNAs.

(B) XTT assay was used to determine the cell proliferation rates in HepG2 and huh7 cells with shNTC or TROY shRNAs.

(C) Analysis of cell distribution in each stage of the cell cycle in each transfected HepG2 HCC cells.

(D) Analysis of cell distribution in each stage of the cell cycle in each transfected Huh7 HCC cells.

(E) XTT assay reveals stronger chemoresistance ability of cells with higher TROY expression compared with that of lower TROY expression in Huh7. Cells were treated with the indicated concentration of Cisplatin (DDP) (48 h) or 5-Fu (48h).

Statistical significances: *, P < 0.05; **, P < 0.01; ***, P < 0.001.

**Supplementary Figure 7**

**
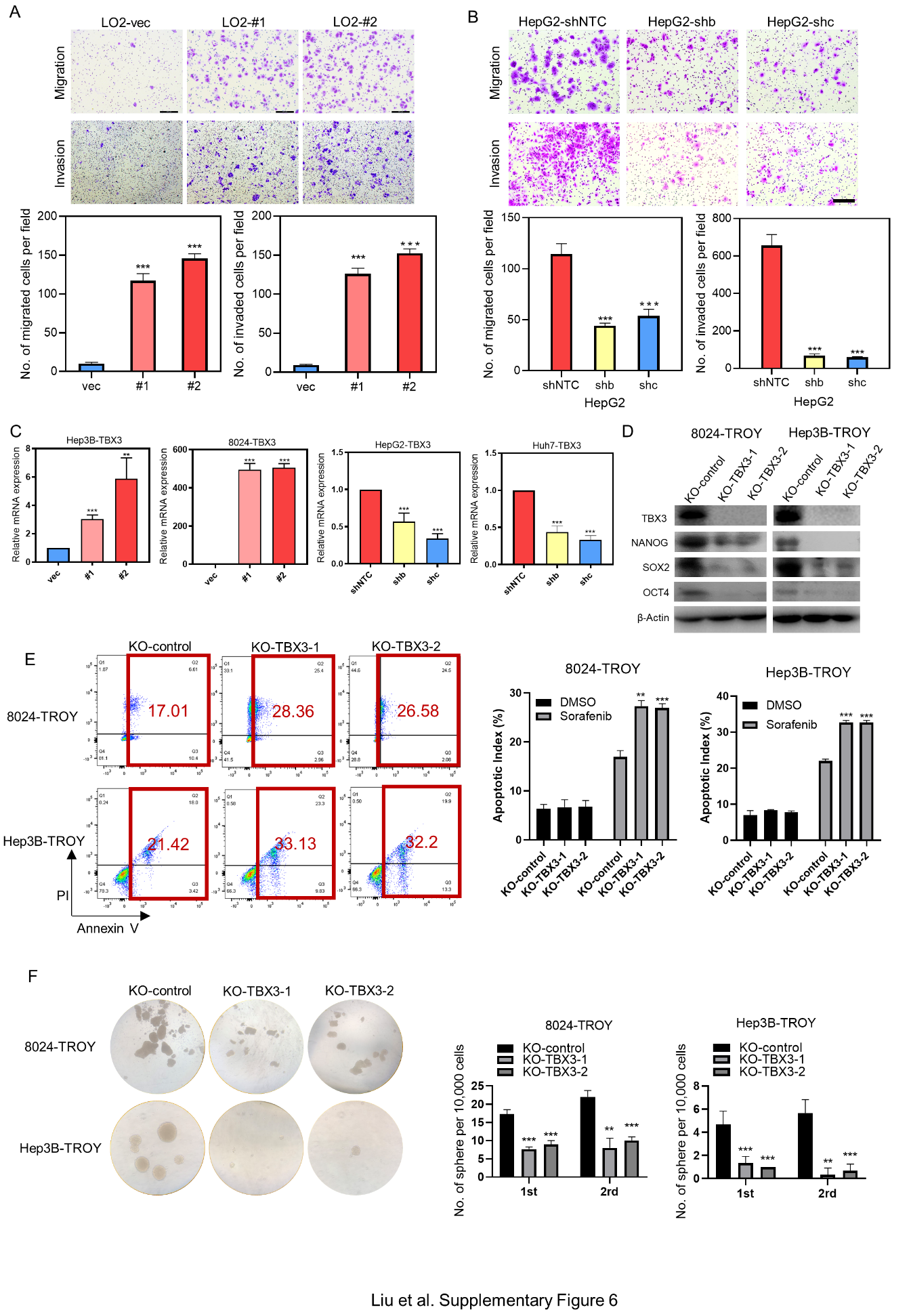
**

(A and B) Transwell migration and matrigel invasion assays and statistics of cell motilities in LO2 cells with vec or TROY isoforms (A) and hepG2 cells with shNTC or TROY shRNAs(B). Scale bar = 200 μm.

(C) qPCR of *TBX3* in 8024 and Hep3B cells transfected with vec or TROY isoforms or HepG2 and huh7 cells with shNTC or TROY shRNAs.

(D) Western blot of TBX3, NANOG, SOX2, OCT3/4 in 8024-TROY and Hep3B-TROY cells with control or TBX3 knockout sgRNAs. β-Actin was used as a loading control in western blot.

(E) Representative flow cytometry plots (left) and summarize(right) of the percentage of apoptotic cells in 8024-TROY and Hep3B-TROY cells with control or TBX3 knockout sgRNAs.

(F) Spheroid formation assay in 8024-TROY and Hep3B-TROY cells with control or TBX3 knockout sgRNAs.

Statistical significances: *, P < 0.05; **, P < 0.01; ***, P < 0.001.

**Supplementary Figure 8**


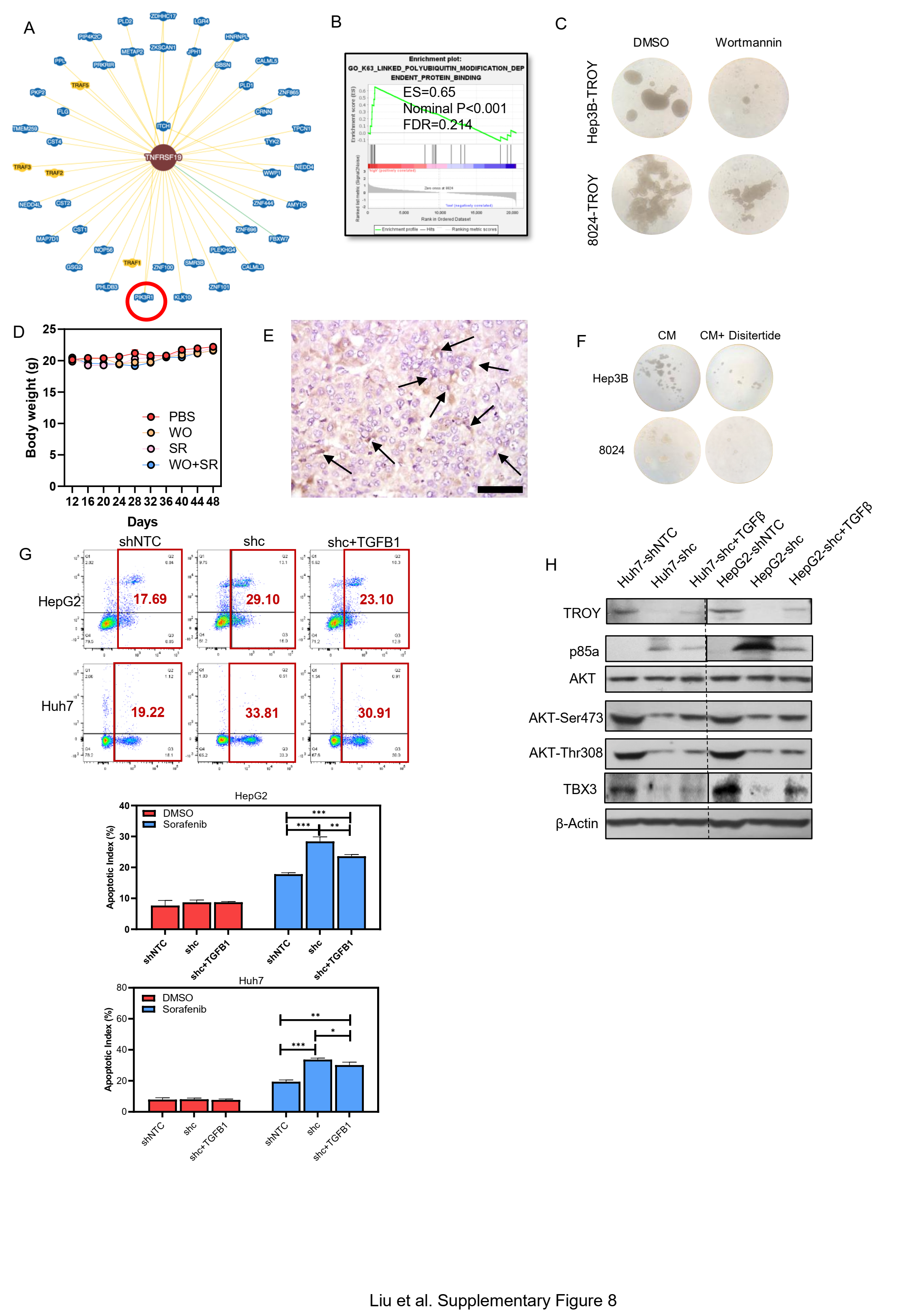


(A) Protein interaction with TROY from BioGRID website.

(B) GSEA analysis showed that high expression of TROY is significantly related to k63 polyubiquitin modification.

(C) Spheroid formation assay in 8024-TROY or Hep3B-TROY cells treated with Wortmannin (50nM) or DMSO.

(D) The body weights of mice in the drug-treated groups remained unchanged.

(E) Representative IHC staining of TGFβ in HCC tissues. TGFβ+ CAF cells were pointed by arrows. Scale bar = 200μm.

(F) Spheroid formation assay in 8024 or Hep3B cells treated with Disitertide (100 μg/mL) or DMSO in CAF conditional medium.

(G) Representative flow cytometry plots of the percentage of apoptotic cells in 8024 or Hep3B cells treated with Disitertide (100 μg/mL) or DMSO in CAF conditional medium.

Western blotting of *TROY*, p85α, Akt, Phospho-Akt (Ser473 and Thr308), and TBX3 in Huh7-shNTC, Huh7-shc, and HepG2-shNTC, HepG2-shc cells treated with PBS (0h) or TGFβ1 (5ng/ml, 24h).

**Supplementary Table 1. Gene cluster A shares a similar expression pattern (correlation co-efficient R>=0.9) both in CK19 and CK7**

| Gene Name | Co-efficient with CK19 | Co-efficient with CK7 |
| --- | --- | --- |
| ZNF703 | 0.984256 | 0.997014 |
| ZNF552 | 0.963595 | 0.951518 |
| XPNPEP1 | 0.982403 | 0.942827 |
| WLS | 0.945285 | 0.922425 |
| WIPI1 | 0.964653 | 0.980196 |
| VTCN1 | 0.976681 | 0.993845 |
| VGLL1 | 0.960401 | 0.978227 |
| UQCR10 | 0.902505 | 0.929699 |
| UBA5 | 0.931809 | 0.926112 |
| TXNDC17 | 0.937344 | 0.916348 |
| TUBB4A | 0.914638 | 0.917983 |
| TSPAN3 | 0.979631 | 0.957754 |
| TSPAN18 | 0.982186 | 0.958747 |
| TRAPPC3 | 0.94963 | 0.939006 |
| TOE1 | 0.9246 | 0.901037 |
| TNFRSF19 | 0.987631 | 0.973776 |
| TMEM41A | 0.982965 | 0.995812 |
| TMEM141 | 0.90903 | 0.916822 |
| TMEM115 | 0.983383 | 0.941617 |
| TJP3 | 0.939995 | 0.974074 |
| TESK2 | 0.927931 | 0.951732 |
| TECR | 0.962599 | 0.966072 |
| TCEB2 | 0.979635 | 0.950118 |
| TBX3 | 0.901267 | 0.941763 |
| TAC3 | 0.976132 | 0.987875 |
| SWI5 | 0.979211 | 0.975045 |
| SUSD2 | 0.981553 | 0.998972 |
| SURF4 | 0.997473 | 0.980823 |
| SULF2 | 0.951194 | 0.900229 |
| STS | 0.911829 | 0.946071 |
| STEAP1 | 0.911391 | 0.943539 |
| SPSB4 | 0.930324 | 0.92024 |
| SP6 | 0.963337 | 0.977939 |
| SOSTDC1 | 0.971 | 0.983457 |
| SOAT2 | 0.980788 | 0.997927 |
| SMTNL2 | 0.973149 | 0.984617 |
| SMAGP | 0.954771 | 0.962055 |
| SLCO4C1 | 0.919958 | 0.92658 |
| SLC43A2 | 0.974464 | 0.987676 |
| SLC35A2 | 0.972024 | 0.933889 |
| SLC2A11 | 0.945698 | 0.94721 |
| SLC25A1 | 0.943757 | 0.976321 |
| SLC22A6 | 0.960371 | 0.97767 |
| SH3KBP1 | 0.962942 | 0.945479 |
| SH3BGRL3 | 0.976955 | 0.956607 |
| SESN3 | 0.919371 | 0.935282 |
| SERPINI1 | 0.947413 | 0.908377 |
| SCUBE3 | 0.989865 | 0.99682 |
| SCOC | 0.967076 | 0.914521 |
| SCD5 | 0.984931 | 0.964547 |
| SCAP | 0.96175 | 0.947463 |
| SAR1A | 0.957383 | 0.911441 |
| S100A16 | 0.975641 | 0.991275 |
| S100A14 | 0.903673 | 0.913767 |
| S100A13 | 0.997798 | 0.988076 |
| S100A11 | 0.984523 | 0.938206 |
| S100A10 | 0.974733 | 0.979999 |
| RSPO3 | 0.952851 | 0.977338 |
| RPL28 | 0.911762 | 0.903172 |
| RP11-47I22.3 | 0.965913 | 0.97971 |
| RNPEP | 0.96805 | 0.930178 |
| RMND1 | 0.902763 | 0.922495 |
| RILPL2 | 0.932115 | 0.952866 |
| RER1 | 0.93267 | 0.946087 |
| RAB4A | 0.930339 | 0.963433 |
| QPRT | 0.917161 | 0.951676 |
| PYGB | 0.993323 | 0.957131 |
| PSTPIP2 | 0.962603 | 0.946231 |
| PRTG | 0.976139 | 0.987716 |
| PRSS12 | 0.97707 | 0.979889 |
| PRR9 | 0.953925 | 0.971709 |
| PRR15 | 0.940279 | 0.947828 |
| PRR13 | 0.98532 | 0.983559 |
| PRKCE | 0.970369 | 0.996948 |
| PNPT1 | 0.95575 | 0.931762 |
| PKP2 | 0.970681 | 0.929274 |
| PITX2 | 0.999235 | 0.979398 |
| PITX1 | 0.9611 | 0.971166 |
| PIGZ | 0.959636 | 0.985445 |
| PIGY | 0.959236 | 0.934469 |
| PGM2 | 0.925778 | 0.914443 |
| PGF | 0.979538 | 0.999371 |
| PFDN5 | 0.953428 | 0.935746 |
| PEX13 | 0.909188 | 0.907482 |
| PCSK1N | 0.959342 | 0.963938 |
| PCP4L1 | 0.924677 | 0.950677 |
| PCDH7 | 0.972887 | 0.908045 |
| PAQR8 | 0.980437 | 0.995594 |
| P2RY6 | 0.979975 | 0.99801 |
| OVOL2 | 0.984548 | 0.96901 |
| OSBPL9 | 0.922524 | 0.933374 |
| NRGN | 0.952171 | 0.97564 |
| NR2F6 | 0.946125 | 0.945007 |
| NPM2 | 0.98346 | 0.979234 |
| NMUR1 | 0.978024 | 0.997559 |
| NLRP7 | 0.963095 | 0.955339 |
| NEU1 | 0.992278 | 0.989693 |
| NAALAD2 | 0.955991 | 0.961316 |
| MYO5B | 0.920654 | 0.951286 |
| MYLIP | 0.954065 | 0.958547 |
| MYL4 | 0.984149 | 0.976122 |
| MYL3 | 0.96973 | 0.989114 |
| MUC15 | 0.953848 | 0.972034 |
| MSX2 | 0.989602 | 0.99548 |
| MRPS36 | 0.956091 | 0.964142 |
| MRPL55 | 0.909729 | 0.932946 |
| MOV10 | 0.966507 | 0.985733 |
| MMP1 | 0.973655 | 0.992774 |
| MGST3 | 0.959795 | 0.981457 |
| MDM1 | 0.938232 | 0.950012 |
| MDK | 0.959067 | 0.931068 |
| MBD1 | 0.962533 | 0.900113 |
| MAN2B1 | 0.920986 | 0.910317 |
| MAGEH1 | 0.95956 | 0.934261 |
| LYPD6B | 0.986354 | 0.993752 |
| LYPD6 | 0.968045 | 0.968839 |
| LRPAP1 | 0.950967 | 0.972746 |
| LRP2 | 0.945217 | 0.955225 |
| LMF2 | 0.906746 | 0.926012 |
| LGALS3 | 0.968382 | 0.990218 |
| LGALS2 | 0.969025 | 0.997038 |
| LGALS16 | 0.954419 | 0.972124 |
| LCP1 | 0.931935 | 0.96477 |
| KRT8 | 0.97681 | 0.927186 |
| KRT7 | 0.978162 | 1 |
| KRT19 | 1 | 0.978162 |
| KRT18 | 0.988756 | 0.95058 |
| KLHDC8B | 0.978026 | 0.950497 |
| KANK4 | 0.974578 | 0.998156 |
| ISCA1 | 0.926305 | 0.910419 |
| IHH | 0.977928 | 0.995886 |
| IGSF1 | 0.982313 | 0.992817 |
| IGDCC3 | 0.97828 | 0.976674 |
| HSPB8 | 0.991881 | 0.969135 |
| HSPA12A | 0.984686 | 0.996577 |
| HOPX | 0.967027 | 0.988128 |
| HLA-DRB5 | 0.961887 | 0.973259 |
| HLA-DRB1 | 0.934202 | 0.977749 |
| HAPLN1 | 0.983898 | 0.998306 |
| HAND1 | 0.974447 | 0.987299 |
| GXYLT2 | 0.946615 | 0.912606 |
| GSN | 0.953958 | 0.971891 |
| GRHL1 | 0.940096 | 0.98083 |
| GOLM1 | 0.918756 | 0.914244 |
| GNPAT | 0.98675 | 0.983359 |
| GNA11 | 0.953221 | 0.910424 |
| GLT25D1 | 0.986275 | 0.943436 |
| GLRX5 | 0.946256 | 0.957236 |
| GLB1 | 0.990057 | 0.989287 |
| GATA3 | 0.987252 | 0.99821 |
| GATA2 | 0.957925 | 0.982252 |
| GABRP | 0.984523 | 0.997675 |
| FRZB | 0.993285 | 0.989577 |
| FREM2 | 0.971986 | 0.973374 |
| FOLR1 | 0.984848 | 0.986031 |
| FMO1 | 0.938867 | 0.969571 |
| FKBP11 | 0.955973 | 0.919629 |
| FEZ2 | 0.936586 | 0.911117 |
| FAM19A4 | 0.97871 | 0.992187 |
| FAM151A | 0.97282 | 0.990955 |
| FAM115A | 0.923276 | 0.922826 |
| EXT2 | 0.977169 | 0.940249 |
| ERVW-1 | 0.965226 | 0.978418 |
| ERVFRD-1 | 0.955621 | 0.97349 |
| ERGIC3 | 0.927865 | 0.912532 |
| EPSTI1 | 0.918357 | 0.904537 |
| EPO | 0.965904 | 0.990384 |
| EPB41L3 | 0.963905 | 0.905781 |
| ELOVL1 | 0.993661 | 0.988628 |
| EIF2B2 | 0.911533 | 0.938601 |
| EFHD1 | 0.961375 | 0.976096 |
| EAPP | 0.959368 | 0.941988 |
| DUSP9 | 0.979253 | 0.997639 |
| DPPA3 | 0.979264 | 0.994372 |
| DPP4 | 0.939768 | 0.981933 |
| DOK4 | 0.965687 | 0.981777 |
| DNAJC15 | 0.978981 | 0.994523 |
| DLX5 | 0.951063 | 0.955214 |
| DHRS11 | 0.97439 | 0.981064 |
| DGAT1 | 0.955331 | 0.990593 |
| DENND2C | 0.92887 | 0.935285 |
| DENND1C | 0.983897 | 0.983484 |
| DDC | 0.900693 | 0.953802 |
| DAP3 | 0.908595 | 0.901325 |
| DACT1 | 0.986098 | 0.956182 |
| CYP4X1 | 0.944473 | 0.979928 |
| CYP19A1 | 0.95325 | 0.971521 |
| CYBA | 0.928208 | 0.903054 |
| CYB5D1 | 0.959606 | 0.957037 |
| CUBN | 0.984822 | 0.997834 |
| CTSE | 0.975575 | 0.992272 |
| CST1 | 0.992479 | 0.947996 |
| CREB3 | 0.986037 | 0.945287 |
| CORO2A | 0.992919 | 0.960639 |
| COL2A1 | 0.970385 | 0.962856 |
| CLDN19 | 0.973745 | 0.985008 |
| CGA | 0.960371 | 0.977811 |
| CDKN1C | 0.914957 | 0.917346 |
| CDK2 | 0.990482 | 0.955757 |
| CDK18 | 0.963001 | 0.987162 |
| CDH1 | 0.932593 | 0.942161 |
| CD99 | 0.963447 | 0.973443 |
| CD63 | 0.97405 | 0.936347 |
| CCBE1 | 0.958977 | 0.98552 |
| CASP6 | 0.957445 | 0.953558 |
| CAMSAP3 | 0.987978 | 0.950168 |
| CAMK2D | 0.978245 | 0.958646 |
| CA4 | 0.971315 | 0.987882 |
| C17orf96 | 0.957942 | 0.974563 |
| C14orf2 | 0.95564 | 0.914737 |
| C11orf24 | 0.926961 | 0.94543 |
| BMP4 | 0.9677 | 0.945804 |
| BIN1 | 0.90928 | 0.931057 |
| BET1L | 0.940745 | 0.931155 |
| BAMBI | 0.986688 | 0.975314 |
| B4GALT1 | 0.965205 | 0.98152 |
| ATP6V0D1 | 0.984704 | 0.950722 |
| ATP6V0A1 | 0.969427 | 0.960066 |
| ATP2B1 | 0.969509 | 0.934999 |
| ARL4C | 0.929827 | 0.969426 |
| ARL2BP | 0.956085 | 0.918763 |
| ARHGAP29 | 0.903494 | 0.911735 |
| ANXA6 | 0.939417 | 0.908193 |
| ANXA2 | 0.980077 | 0.919903 |
| ANKMY2 | 0.983344 | 0.93092 |
| AMOT | 0.986496 | 0.996035 |
| AMN | 0.942839 | 0.97366 |
| AKAP5 | 0.962721 | 0.947623 |
| AK4 | 0.92022 | 0.929294 |
| AGPHD1 | 0.913591 | 0.947265 |
| ADD3 | 0.985235 | 0.965672 |
| ADAMTS18 | 0.981695 | 0.967794 |
| ACTC1 | 0.980482 | 0.920281 |
| ABHD5 | 0.995992 | 0.966669 |

**Supplementary Table 2. Frequencies of TROY-expression cells in HCC tumor tissues**

| **TROY-expression cells (%)** | **No. of HCC cases** |
| --- | --- |
| 0-0.25 | 42 |
| 0.25-0.5 | 35 |
| 0.5-1 | 22 |
| 1-2 | 15 |
| 2-3 | 10 |
| 3-4 | 4 |
| 4-5 | 2 |
| **Total** | **130** |

**Supplementary Table 3 Association between TROY expression and clinicopathologic features in 130 HCC cases.**

| **Features** | **Total** | **TROY expression^a^** | | ***P*-value** |
| --- | --- | --- | --- | --- |
|  |  | **negative** | **Positive** |  |
| *Sex* |  |  |  | 0.365 |
| Male | 123 | 74 | 49 |  |
| Female | 7 | 3 | 4 |  |
| *Age (years)* |  |  |  | **0.027** |
| ≤60 | 100 | 54 | 46 |  |
| >60 | 30 | 23 | 7 |  |
| *Serum AFP (ng/mL)* |  |  |  | 0.526 |
| ≤400 | 73 | 45 | 28 |  |
| >400 | 57 | 32 | 25 |  |
| *Serum HBsAg* |  |  |  | 0.622 |
| Negative | 17 | 11 | 6 |  |
| Positive | 113 | 65 | 48 |  |
| *Cirrhosis* |  |  |  | 0.237 |
| Absent | 21 | 10 | 11 |  |
| Present | 109 | 67 | 42 |  |
| *Tumor size* |  |  |  | **0.024** |
| ≤5 | 52 | 37 | 15 |  |
| >5 | 78 | 40 | 38 |  |
| *TNM stage (AJCC)* |  |  |  | 0.835 |
| I/ II | 75 | 45 | 30 |  |
| III/IV | 55 | 32 | 23 |  |
| *Invasion* |  |  |  |  |
| Absent | 98 | 59 | 39 | 0.693 |
| Present | 32 | 18 | 14 |  |
| *Metastasis* |  |  |  | **0.042** |
| Absent | 89 | 58 | 31 |  |
| Present | 41 | 19 | 22 |  |

1. Pearson *χ*^2^ test.
2. Statistical significance (*P* < 0.05) is shown in bold.
3. #Partial data are not available, and the statistic was based on available.
4. ^a^TROY positive: Samples with high-frequency of TROY (TROY*^+^*, > 0.5%, n = 53) detection by IHC staining; Samples with low-frequency of TROY (TROY*^-^*, ≤ 0.5%, n = 77) detection by IHC staining.

**Supplementary Table 4. Enriched KEGG pathway of gene up-regulated from the 20 *TROY^hi^* patients.**

| Term | Count | PValue | Pop Hits | Fold Enrichment |
| --- | --- | --- | --- | --- |
| hsa05200:Pathways in cancer | 36 | 2.29E-06 | 393 | 2.368938 |
| hsa04151:PI3K-Akt signaling pathway | 27 | 7.36E-04 | 345 | 2.023897 |
| hsa04310:Wnt signaling pathway | 25 | 3.49E-10 | 138 | 4.684946 |
| hsa04510:Focal adhesion | 24 | 3.64E-06 | 206 | 3.012921 |
| hsa04024:cAMP signaling pathway | 19 | 5.86E-04 | 198 | 2.481602 |
| hsa05205:Proteoglycans in cancer | 19 | 6.60E-04 | 200 | 2.456786 |
| hsa04010:MAPK signaling pathway | 19 | 0.008605 | 253 | 1.942123 |
| hsa04020:Calcium signaling pathway | 18 | 5.03E-04 | 179 | 2.600538 |
| hsa04080:Neuroactive ligand-receptor interaction | 18 | 0.037943 | 277 | 1.680492 |
| hsa04974:Protein digestion and absorption | 16 | 1.04E-06 | 88 | 4.701982 |
| hsa04390:Hippo signaling pathway | 16 | 6.70E-04 | 151 | 2.740228 |
| hsa04810:Regulation of actin cytoskeleton | 16 | 0.015188 | 210 | 1.970354 |
| hsa04512:ECM-receptor interaction | 15 | 4.88E-06 | 87 | 4.458776 |
| hsa04261:Adrenergic signaling in cardiomyocytes | 15 | 8.18E-04 | 138 | 2.810968 |
| hsa04921:Oxytocin signaling pathway | 15 | 0.00184 | 150 | 2.58609 |
| hsa05146:Amoebiasis | 14 | 1.97E-04 | 106 | 3.415591 |
| hsa04022:cGMP-PKG signaling pathway | 14 | 0.007745 | 158 | 2.291472 |
| hsa04550:Signaling pathways regulating pluripotency of stem cells | 13 | 0.007552 | 140 | 2.401369 |
| hsa05412:Arrhythmogenic right ventricular cardiomyopathy (ARVC) | 12 | 4.25E-05 | 67 | 4.631803 |
| hsa05204:Chemical carcinogenesis | 12 | 2.22E-04 | 80 | 3.879135 |
| hsa05217:Basal cell carcinoma | 11 | 3.24E-05 | 54 | 5.267962 |
| hsa05410:Hypertrophic cardiomyopathy (HCM) | 11 | 7.55E-04 | 78 | 3.64705 |
| hsa05414:Dilated cardiomyopathy | 11 | 0.001353 | 84 | 3.386547 |
| hsa04911:Insulin secretion | 11 | 0.001483 | 85 | 3.346705 |
| hsa04725:Cholinergic synapse | 11 | 0.010197 | 111 | 2.562792 |
| hsa04611:Platelet activation | 11 | 0.027995 | 130 | 2.18823 |
| hsa00982:Drug metabolism - cytochrome P450 | 10 | 0.001095 | 68 | 3.803074 |
| hsa04976:Bile secretion | 10 | 0.001218 | 69 | 3.747957 |
| hsa00980:Metabolism of xenobiotics by cytochrome P450 | 10 | 0.002009 | 74 | 3.494717 |
| hsa05222:Small cell lung cancer | 10 | 0.005183 | 85 | 3.042459 |
| hsa04972:Pancreatic secretion | 10 | 0.009281 | 93 | 2.780742 |
| hsa04916:Melanogenesis | 10 | 0.014555 | 100 | 2.58609 |
| hsa04360:Axon guidance | 10 | 0.055858 | 127 | 2.036292 |
| hsa04514:Cell adhesion molecules (CAMs) | 10 | 0.096748 | 142 | 1.82119 |
| hsa05218:Melanoma | 9 | 0.005668 | 71 | 3.278143 |
| hsa04260:Cardiac muscle contraction | 9 | 0.007878 | 75 | 3.103308 |
| hsa05215:Prostate cancer | 9 | 0.0196 | 88 | 2.644865 |
| hsa05032:Morphine addiction | 9 | 0.023499 | 91 | 2.557672 |
| hsa05231:Choline metabolism in cancer | 9 | 0.040375 | 101 | 2.304437 |
| hsa05145:Toxoplasmosis | 9 | 0.061205 | 110 | 2.115892 |
| hsa04670:Leukocyte transendothelial migration | 9 | 0.075244 | 115 | 2.023897 |
| hsa04919:Thyroid hormone signaling pathway | 9 | 0.075244 | 115 | 2.023897 |
| hsa00830:Retinol metabolism | 8 | 0.011075 | 64 | 3.232613 |
| hsa04970:Salivary secretion | 8 | 0.04722 | 86 | 2.405665 |
| hsa04540:Gap junction | 8 | 0.052345 | 88 | 2.350991 |
| hsa04713:Circadian entrainment | 8 | 0.07296 | 95 | 2.17776 |
| hsa00140:Steroid hormone biosynthesis | 7 | 0.023376 | 58 | 3.121143 |
| hsa04971:Gastric acid secretion | 7 | 0.061378 | 73 | 2.479813 |
| hsa04925:Aldosterone synthesis and secretion | 7 | 0.091276 | 81 | 2.234893 |
| hsa05210:Colorectal cancer | 6 | 0.089537 | 62 | 2.502668 |
| hsa04964:Proximal tubule bicarbonate reclamation | 5 | 0.01069 | 23 | 5.621935 |
| hsa00350:Tyrosine metabolism | 5 | 0.044182 | 35 | 3.694415 |
| hsa00910:Nitrogen metabolism | 4 | 0.025754 | 17 | 6.084918 |
| hsa00532:Glycosaminoglycan biosynthesis - chondroitin sulfate / dermatan sulfate | 4 | 0.039681 | 20 | 5.17218 |
| hsa00534:Glycosaminoglycan biosynthesis - heparan sulfate / heparin | 4 | 0.063006 | 24 | 4.31015 |

**Supplementary Table 5. List of primers used in this study.**

| Gene | Primer | Sequence |
| --- | --- | --- |
| For Real-time PCR  TROY-isoform1 | Forward | GGAGAGTGGTGCTGTCATCC |
|  | Reverse | CCCCCAAATCTTATGCTGGGA |
| TROY-isoform2 | Forward | GCAGCTGTAACTGCCCTAGT |
|  | Reverse | ATGGTAGGGGATGGAGCACT |
| TROY | Forward | TGCTTGCCAGGATTTTATAGGAA |
|  | Reverse | GACGCGATCTTCACGAGGTT |
| NANOG | Forward | CCCCAGCCTTTACTCTTCCTA |
|  | Reverse | CCAGGTTGAATTGTTCCAGGTC |
| SOX2 | Forward | TACAGCATGTCCTACTCGCAG |
|  | Reverse | GAGGAAGAGGTAACCACAGGG |
| OCT4 | Forward | CTTGAATCCCGAATGGAAAGGG |
|  | Reverse | GTGTATATCCCAGGGTGATCCTC |
| TBX3 | Forward | GAAGCCTGTTCCCTTACCCC |
|  | Reverse | GGGATGGAGTAGGGGCTGTA |
| For clone gene  Knockout-p85α | Forward | CACCGTGATTATACTCTTACACTA |
|  | Reverse | AAACTAGTGTAAGAGTATAATCAC |
| Knockout-TBX3-1 | Forward | CACCGTGGGAGGCAGCGTCAGCGCG |
|  | Reverse | AAACCGCGCTGACGCTGCCTCCCA |
| Knockout-TBX3-2 | Forward | CACCGGAGAACGGGATGCCGGTCT |
|  | Reverse | AAACAGACCGGCATCCCGTTCTCC |
| TROY-isoform1 | Forward | TACTCTAGAGCTAGCGAATTCGCCACCATGGCTTTAAAAGTGCTACT |
|  | Reverse | GAGGGGCGCGGCCGCGGATCCATCTACTGTAAGTCAGTGCTGTGT |
| TROY-isoform2 | Forward | TACTCTAGAGCTAGCGAATTCGCCACCATGGCTTTAAAAGTGCTACTA |
|  | Reverse | GAGGGGCGCGGCCGCGGATCCCTCTTTGGGTTCCAGCACAC |

**Supplementary Table 6. List of antibodies used in this study.**

| Antibody | Size (KDa） | Vendor | Cat No. | Application |
| --- | --- | --- | --- | --- |
| Mouse anti-TROY | 45 | Santa Cruz | sc-398526 | IP, 1: 50; IF,1:100 |
| Rabbit anti-TROY | 46 | Abcam | ab138502 | IHC, 1:100; Flow Cyt, 1:100; WB, 1:1000 |
| Mouse anti-p85α | 85 | Santa Cruz | sc-1637 | IP, 1: 50; WB, 1:1000; IF,1:100; IHC, 1:100 |
| Rabbit anti-Akt | 60 | Cell signaling | CST4691 | WB, 1:1000 |
| Rabbit anti-phospho Akt (Ser473) | 60 | Cell signaling | CST9271 | WB, 1:1000 |
| Rabbit anti-phospho Akt (T308) | 60 | Abcam | ab38449 | WB, 1:1000 |
| Rabbit anti-TBX3 | 79 | Abcam | ab99302 | WB, 1:1000 |
| Rabbit anti-NANOG | 42 | Cell signaling | CST4903 | WB, 1:1000 |
| Rabbit anti-SOX2 | 34 | Abcam | ab92494 | WB, 1:1000 |
| Mouse anti-OCT4 | 39 | Abcam | ab184665 | WB, 1:1000 |
| Rabbit anti-E-cadherin | 135 | Cell signaling | CST3195 | WB, 1:1000 |
| Rabbit anti-β-catenin | 92 | Cell signaling | CST9562 | WB, 1:1000 |
| Rabbit anti-N-cadherin | 140 | Cell signaling | CST4061 | WB, 1:1000 |
| Rabbit anti-Vimentin | 57 | Cell signaling | CST5741 | WB, 1:1000 |
| Mouse anti-β-actin | 43 | Abcam | ab6276 | WB, 1:5000 |
